# Caffeine attenuates cisplatin induced microglial reactivity and cognitive dysfunction

**DOI:** 10.3389/fnmol.2026.1772430

**Published:** 2026-05-13

**Authors:** Alfredo Oliveros, Ivan Loncar, Marwan Mostafa, Michael Poleschuk, Ana M. Corujo, Bo Qin, Juhyun Song, Max A. Tischfield, Mi-Hyeon Jang

**Affiliations:** 1 Department of Neurosurgery, Robert Wood Johnson Medical School, Rutgers University, Piscataway, NJ, United States; 2 Department of Biological Sciences, The State University of New York at Buffalo, Buffalo, NY, United States; 3 Department of Neurological Surgery, Mayo Clinic College of Medicine, Rochester, MN, United States; 4 Section of Cancer Epidemiology and Health Outcomes, Rutgers Cancer Institute, New Brunswick, NJ, United States; 5 Department of Anatomy, Chonnam National University Medical School, Hwasun, Republic of Korea; 6 Department of Cell Biology and Neuroscience, School of Arts and Sciences, Rutgers University, Piscataway, NJ, United States

**Keywords:** adenosine receptor 2A, caffeine, chemobrain, chemofog, cisplatin, microglia, neurogenesis

## Abstract

Advances in cancer treatment by chemotherapy, radiation, and personalized targeted therapy have significantly improved cancer survivorship. Life extension from chemotherapy is arguably the most accessible non-surgical treatment strategy, but it is accompanied by neurotoxic sequelae that are detrimental to cognitive function in survivors. Using the platinum-based drug cisplatin to model chemotherapy induced cognitive impairments (CICI; also called chemobrain) in mice, we recently identified that elevated hippocampal adenosine A_2A_ receptor (A_2A_R) levels are causally associated with CICI. Although neuroinflammation is a major pathological mechanism underlying CICI, it remains unclear whether A_2A_R mediates cisplatin-induced neuroinflammation. To address this knowledge gap, we demonstrate for the first time that cisplatin increases microglial process extension and branching complexity, consistent with a primed, hyper-surveillant morphological phenotype. Remarkably, caffeine, a non-specific A_2A_R antagonist and known cognitive enhancer, attenuated the primed microglial morphology caused by cisplatin. Furthermore, we show that caffeine mitigates cisplatin-induced body-weight loss, accelerates physical recovery, and protects against motor dysfunction. We also show that caffeine confers protection against cisplatin potentiated impairments in hippocampal dendritic spine density, neurogenesis, and confers modest memory benefits. Collectively, these results suggest that A_2A_R-mediated microglial priming is associated with cognitive dysfunction in chemobrain and A_2A_R inhibition by caffeine may represent a strategy to ameliorate the physical and cognitive impairments caused by cisplatin.

## 1 Introduction

Survivorship data from the Surveillance, Epidemiology, and End Results (SEER) program reports more than 18 million cancer survivors in the United States, a number estimated to increase to 22.1 million by 2030 (Miller et al., 2022). Chemotherapy-induced cognitive impairment (CICI) is common in 50%–75% of acutely treated cancer patients, and may persist months-to-years in 35% of survivors (Mayo et al., 2021). Clinically, chemotherapy impairs attention, processing speed, and memory, and also potentiates body-weight loss (Lange et al., 2019; Lustberg et al., 2023; Nevins et al., 2023). Platinum-based compounds such as cisplatin have been widely used to treat prostate, ovarian and breast cancer; however, their neurotoxicity decreases quality of life (QoL) in survivors (Balmana et al., 2014; Amidi et al., 2017; Smith et al., 2024).

We previously demonstrated that cisplatin specifically elevates adenosine A_2A_ receptor (A_2A_R) expression in the hippocampus, a brain region that is critical for memory and mood (Oliveros et al., 2022). In that report, we further showed that A_2A_R inhibition with istradefylline attenuated cisplatin-induced neurogenesis impairment and memory dysfunction. Adenosine exerts its neuromodulatory effects through four G-protein coupled adenosine A_1_, A_2A_, A_2B_, and A_3_ receptor subtypes (Burnstock, 2020). Caffeine, a non-specific antagonist of A_2A_R, improves synaptic plasticity and cognitive function in normal aging, as well as preclinical and clinical investigations of Alzheimer’s disease (AD) (Canas et al., 2009). Further, pharmacological activation, or genetic overexpression of A_2A_R, results in impaired cognitive performance in mice (Pereira et al., 2005; Gimenez-Llort et al., 2007; Li et al., 2015), whereas genetic A_2A_R deletion or pharmacological A_2A_R antagonism improve cognitive performance (Wang et al., 2006; Wei et al., 2011; Pagnussat et al., 2015; Orr et al., 2018).

Interestingly, A_2A_R also contributes to neuroinflammatory responses, as well as process extension dynamics of microglia, the brain-resident immune cell (Canas et al., 2009; Orr et al., 2009; Chen et al., 2018). Neuroinflammation in CICI is being explored as an etiological mechanism, given that chemotherapy activates brain microglia (Gibson et al., 2019; Subramaniam et al., 2022; Wen et al., 2022), whereas reduced brain volumes and increased circulating proinflammatory cytokines are found in chemotherapy treated cancer survivors (Phillips et al., 2020; Belcher et al., 2022).

Given that cisplatin results in genotoxicity-related peripheral inflammation (de Biasi et al., 2014), coupled with A_2A_R’s role in control of cognition, here we report that the A_2A_R antagonist caffeine attenuates neuroinflammatory microglial activation, impaired dendritic spine density, and reduced neurogenesis in the hippocampus following cisplatin treatment. Concomitantly, caffeine has modest protective benefits against cisplatin-induced memory dysfunction and mitigates cisplatin-induced physical detriments. Taken together, we propose that A_2A_R inhibition by caffeine may represent a novel strategy against physical and cognitive impairments derived from chemotherapy.

## 2 Materials and methods

Detailed methods are provided in the accompanying Supplementary Information.

### 2.1 Mouse husbandry and drug treatment

All experiments were performed on 3–4 months-old female C57BL/6J mice (Jackson Laboratory), housed in a standard ventilated cage system under a 12-h light/dark cycle. Water and food were provided *ad libitum* in the home cage. Mice were randomly allocated to each treatment group prior to the onset of treatments and identification of each animals was done via marks. Tail marks were repeated as necessary to reliably identify animals until experimental endpoints. For behavior experiments, mice were habituated for at least 30 min to the behavior testing room and mice underwent testing starting at 1300 h. All experimental procedures were approved by Institutional Animal Care and Use Committee’s at the Mayo Clinic, and Rutgers University.

Cisplatin treatment regimen: in our studies, one treatment cycle consists of a once daily cisplatin injection (2.3 mg/kg i.p./day, dissolved in 0.9% saline, delivered at 0.1 mL/10 g body weight; Fresenius Kabi, Lake Zurich IL, USA) or vehicle injection (0.9% saline), administered for 5 consecutive days, followed by 5 days of rest from injections, and this treatment schedule was repeated for 3-cycles (Yoo et al., 2021; Oliveros et al., 2022). This dosing regimen of cisplatin has been previously demonstrated to pathologically accumulate in dorsal root ganglia neurons to exert peripheral neuropathies (Gill and Windebank, 1998; Ta et al., 2009), as well as potentiate neurocellular and cognitive dysfunction akin to clinical cognitive impairments (Le and Hanna, 2018; Szturz et al., 2019).

**FIGURE 1:**
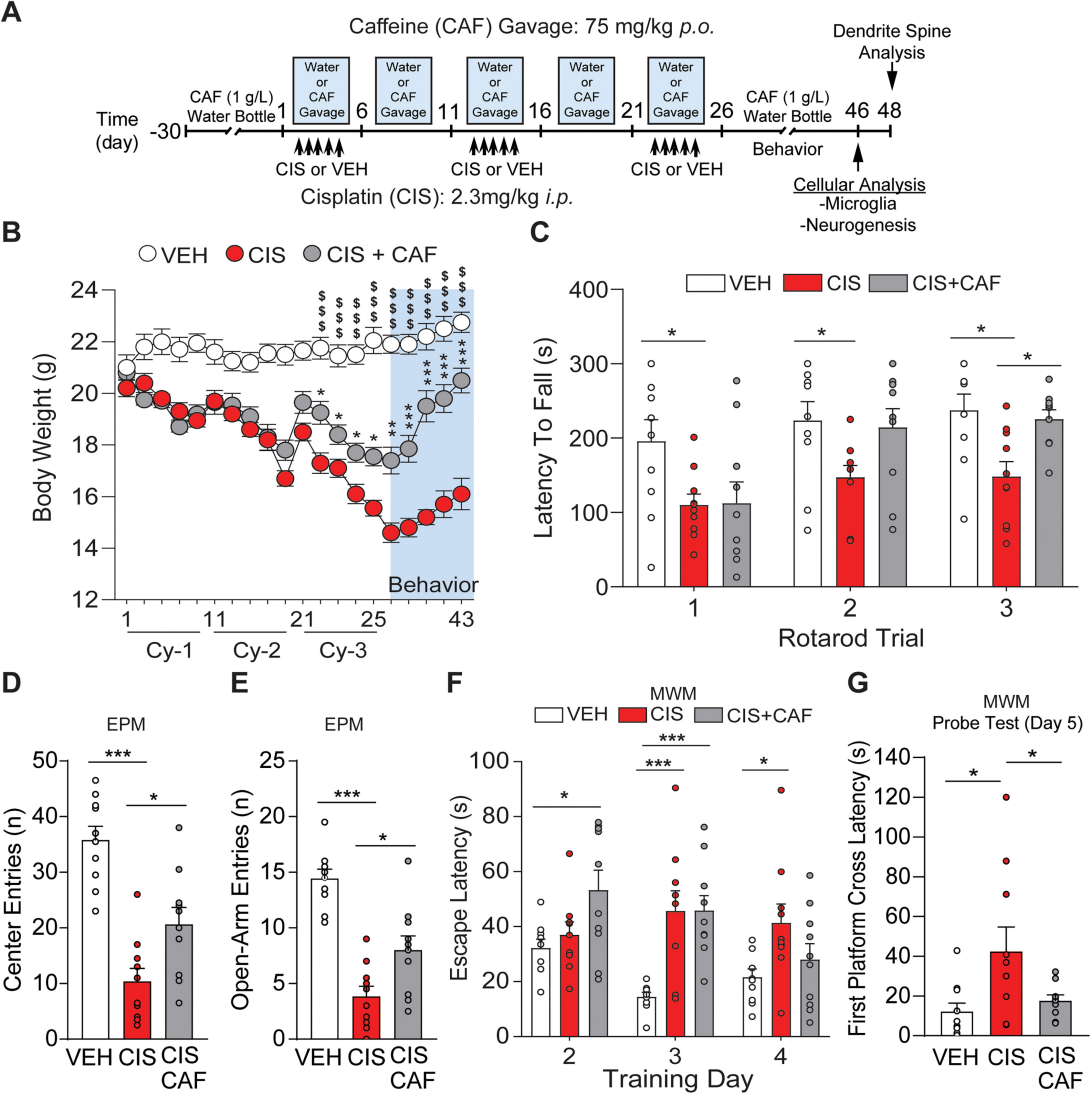
Chronic caffeine exposure prevents cisplatin induced deficits in physicality and cognition. **(A)** Timeline of caffeine pretreatment in the drinking water (1 g/L; free drinking condition), followed by oral gavage of water or caffeine-alone (CAF; 75 mg/kg, *p.o.*), or oral gavage of caffeine plus injections of cisplatin (CIS+CAF), oral gavage of water plus cisplatin-alone injections (CIS; 2.3 mg/kg *i.p.*) or oral gavage of water plus vehicle-alone (VEH) injections. Timeline also shows follow up behavior assays, and cellular analysis. Upward arrows indicate daily consecutive CIS injections. **(B)** Body-weight analysis across 3-cycles of treatment and during behavior testing indicates improved body-weight by caffeine in CIS+CAF treated mice when compared to CIS treated mice. **(C)** Compared to CIS treatment, CIS+CAF treated mice demonstrate improved motor performance during the accelerated Rotarod. **(D)** Analysis of elevated plus maze (EPM) center entries, and **(E)** open-arm entries, show that CIS+CAF treated mice entered these exposed sections of the maze more frequently in comparison to CIS treated mice. **(F)** During MWM spatial memory training, CIS treated mice exhibited significantly longer latencies to find the hidden platform on Day 3 and Day 4, when compared to VEH, indicative of impaired spatial learning by CIS. **(G)** Memory Probe test (Day 5): quantitative analysis of latency to the first platform cross from mice released from the NE point shows that VEH and CIS+CAF treated mice exhibited significantly more platform crossings (in SE quadrant) when compared to CIS alone treated mice. **(B)** Repeated measures (RM) 2-way ANOVA, Tukey’s *post hoc*. *n* = 10 mice/treatment group. **(C)** RM 2-way ANOVA, Holm Sidak’s *post hoc*. *n* = 10 mice/treatment group. **(D,E)** 1-way ANOVA, Tukey’s *post hoc*. *n* = 10 mice/treatment group. **(F)** RM 2-way ANOVA, Tukey’s *post hoc*. *n* = 9–10 mice/treatment group. **(G)** 1–way ANOVA, Holm-Sidak *post hoc*. *n* = 9–10 mice/treatment group. *: *P* < 0.05, ***: *P* < 0.001. Results are reported in mean ± SEM.

Caffeine regimen prior to cisplatin treatment cycles: cisplatin’s half-life in the mouse circulatory compartment is reported to be 15–33 min (Daley-Yates and McBrien, 1984; Gondal et al., 1993). However, it readily distributes and persists in malignant and normal tissues beyond 24 h (Breglio et al., 2017; Perse, 2021), including the hippocampus (Nakagawa et al., 1996; Yoo et al., 2021). Given cisplatin’s persistence in normal-and-malignant tissues, we established chronic caffeine consumption as a prophylactic approach prior to cisplatin treatments by adding caffeine in the drinking water bottle (1 g/L; MilliporeSigma; Figure 1A, Supplementary Figure 4A, and Supplementary Table 1). Although considered a high dose, 1 g/L chronic caffeine intake provided in this free drinking condition has been shown to promote rewards motivated behavior (Hinton et al., 2019). More “in the line “293” for clarity.importantly, 1 g/L chronic caffeine provided in the drinking water exerts neuroprotective effects against cognitive impairments exerted by various conditions, including diabetes (Duarte et al., 2009), Kainic acid induced convulsions (Cognato et al., 2010), and chronic unpredictable stress (Kaster et al., 2015). Caffeine also increases BDNF (Ardais et al., 2014), which is known to preserve synaptic integrity and cognitive function, without causing neuroinflammatory reactive astrogliosis. When caffeine was provided in the drinking water prior to cisplatin treatment, water delivery in the ventilated rack housing system was disconnected. Caffeine regimen during cisplatin treatment cycles: caffeine is a known diuretic that has a bitter taste, can lower water intake, and generate conditioned taste aversion in rodents (White and Mason, 1985; Nagatomo and Kubo, 2008; Seal et al., 2017). Moreover, given that cisplatin can detrimentally reduce water intake (Perse, 2021), to ensure that our cisplatin treated cohorts had adequate hydration, we adapted our caffeine administration strategy from caffeine in the drinking water (free drinking condition), to administration of caffeine via oral (p.o.) gavage (Cat# 18061-22; Fine Science Tools, Foster City CA, USA). Consequently, during cisplatin or vehicle treatment cycles, all mice had *ad-libitum* access to water via the home cage rack system (Supplementary Table 1). The caffeine dose for Experiment 1 was at 75 mg/kg/day and the caffeine dose for Experiment 2 was 37.5 mg/kg/day (Figure 1A, Supplementary Figure 4A, and Supplementary Table 1). Given the accumulation of cisplatin in the brain, we hypothesized that these doses would maintain elevated caffeine levels during treatment cycles, thus protecting against cisplatin’s negative effects on brain function. In mice, a 15 mg/kg dose yields approximately peak levels of 15 μg/mL caffeine in the circulatory compartment, which is relatively similar to 500 mg (18 μg/mL) in the circulatory compartment from a 20 oz serving of caffeine in humans, although its half-life in the mouse blood and brain is decisively shorter, at around 1.6 h (Kaplan et al., 1989, 1990; May et al., 2015). Consequently, we administered a once per day caffeine dose of 75 mg/kg/day (3.75 mg/mL, p.o.) or water (dose/volume: 0.2 mL/10 g body-weight), 1 h prior to cisplatin injections to maintain elevated levels of caffeine during cisplatin treatment cycles. This strategy avoided the stress that multiple, daily, oral gavages would have in our mice. Interestingly, our selected dose of caffeine is within the range of doses (60–100 mg/kg) shown to be protective against lethal doses of radiation induced weight loss, hormone depletion, and elevated gene expression of pro-apoptotic and DNA damage response mediators (George et al., 1999; Kim et al., 2003, 2005). During the 5-days rest from injections, mice continued to receive caffeine or water via oral gavage to avoid caffeine withdrawal (Georgiev et al., 1993; Mahdi et al., 2019). Following the completion of 3 cycles of treatment, we ceased to provide caffeine or water via oral gavage due to the stress of this procedure (Figure 1A, Supplementary Figure 4A, and Supplementary Table 1).

### 2.2 Body-weight measurements

Physiological frailty is a common phenotype experienced by adult and pediatric cancer survivors administered chemotherapy (Ness et al., 2013; Demaria et al., 2017; Wang et al., 2021), including survivors treated with cisplatin (Moreira-Pais et al., 2018). Our previous study indicated that A_2A_R antagonism by the FDA approved drug istradefylline significantly improved body weight recovery from cisplatin chemotherapy (Oliveros et al., 2022). We used body weight as a determinant of health in our mouse cohorts to detect a frailty-like phenotype resulting from chemotherapy. Our selection of mice at 3–4 months of age ensured that body weights were sufficiently robust prior to cisplatin treatment and none of the treatment groups had significantly different weights on the day before the onset of treatment cycles (Day 0; Supplementary Figures 1A, 4C).

### 2.3 Behavioral analysis

All mice underwent acclimation to the behavior testing room for 1-h prior testing to minimize stress. All behavior tests were video recorded. Tracking analysis was performed with Noldus EthoVision-XT. Elevated plus maze: mice were released at the center location and allowed to freely explore the open and closed arms of the maze for 5 min (Supplementary Figure 1A). Distance traveled, time spent in each maze location, latency to 1st center entry, and frequency of entries to each maze location were analyzed (Walf and Frye, 2007). Morris water maze: briefly, the MWM is a hippocampal dependent assay where mice learn to use spatial cues surrounding a circular tank to escape onto a hidden-submerged platform (Vorhees and Williams, 2006). Four spatial cues were placed at North (N), South (S), East (E), and West (W) cardinal points, dividing the tank into four quadrants. Mice were released from NE, SE, SW, NW points (Supplementary Figure 1D) and each release trial had a maximum duration of 2 min. Latency to escape onto the hidden-submerged platform was used as our measure of learning. The test was performed across 6 consecutive days consisting of visible platform days (Day 1, Day 6). Visible platform day (Day 1 and Day 6): maximum of 2 min/trial for each release point (NE→SE→SW→NW). If an animal found the platform within the 2 min trial, they were allowed to remain there for 30 s before being removed to await the next trial (i.e., next release point). Failure to find platform: mice were guided to platform and remained there for 30 s. Training (Days 2–4): the flag was removed, and mice relied on spatial cues to learn to escape onto the submerged platform with a maximum of 2 min/trial for each release point. If mice found the platform in less than 2 min, the trial ended. If mice were unable to escape onto the platform within 2 min, they were guided to swim to the platform and allowed to remain there for an additional 30 s. Swim speed and escape latencies were measured for Days 1–4, and Day 6. Memory Probe test (Day 5): The platform was removed, with a maximum of 2 min/trial. All measurements from the Memory Probe test are from the first release point (NE) release Trial (2 min) point. During the Probe test, latency to the first platform cross, frequency of platform crosses, latency to the first SE quadrant cross, % time spent in the SE quadrant and swim speed were measured. Y-maze: we assessed working memory by spontaneous alternation in the Y-maze (Hughes, 2004). Operationally, three identical equidistant arms were designated A, B, C, and correct alternations were defined as sequential entries into arms (e.g., C→A→B, or B→A→C). Mice were allowed to freely explore the maze for 5-min. Arm entries, distance traveled, and alternations were measured. Percentage alternation was calculated using the following equation:


(C⁢o⁢r⁢r⁢e⁢c⁢t⁢A⁢l⁢t⁢e⁢r⁢n⁢a⁢t⁢i⁢o⁢n⁢sM⁢a⁢x⁢i⁢m⁢u⁢m⁢A⁢l⁢t⁢e⁢r⁢n⁢a⁢t⁢i⁢o⁢n⁢s)⁢x⁢ 100

Accelerated Rotarod: mice underwent testing across three trials separated by 30 min between each trial, using a Rotarod treadmill (Ugo Basile), programmed to gradually accelerate from a 2-rpm to 40-rpm over a 300 s period (Oliveros et al., 2017). Latency-to-fall from the rotating beam was our measure of physical function.

### 2.4 Brain preparation, imaging and analysis

**FIGURE 2:**
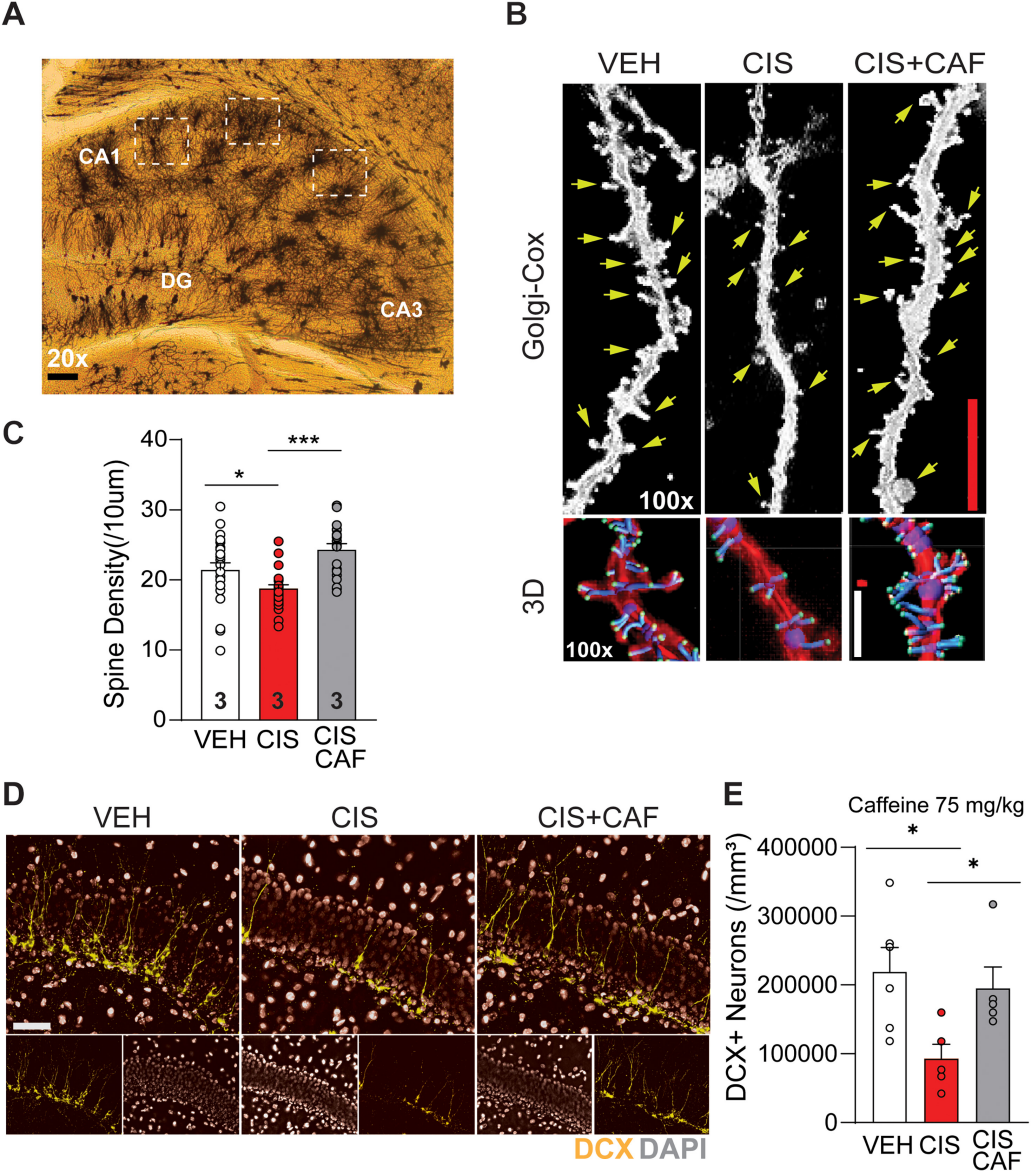
Caffeine protects against cisplatin-induced dendrite spine density impairments and neurogenesis in the hippocampus. **(A)** Representative micrograph of Golgi-Cox stained neurons from vehicle (VEH) treated mice indicates the regions of interest (white dashed squares) selected for dendrite spine analysis in the hippocampal CA1 subregion. DG, dentate gyrus; CA, Cornu *Ammonis*. Scale bar: 200 μm. 20× magnification. **(B)** Representative light microscopy images (100× magnification) of Golgi-Cox stained basal secondary branch dendrites shows dendrite spines (green arrows) from pyramidal neurons in the CA1 subregion of the hippocampus in mice administered VEH, 3-cycles of cisplatin (CIS; 2.3 mg/kg, *i.p.*), or mice that were pretreated with caffeine (75 mg/kg, *p.o.*) in combination with cisplatin (CIS+CAF). Scale bar (red): 5 μm. Representative images of 3D reconstruction of Golgi-Cox stained hippocampal dendrite spines from VEH, CIS, and CIS+CAF treated brains. Scale bar (white): 2.5 μm. **(C)** Quantitative analysis of 3D reconstructed dendrite spines shows that 3-cycles of CIS (*n* = 26 dendrites shown across 5–11 neurons/brain) administration significantly reduces dendrite spine densities when compared to VEH (*n* = 26 dendrites shown 6–11 neurons/brain) and CIS+CAF (*n* = 20 dendrites shown 6–7 neurons/brain) treated mice. One-way ANOVA, Dunnett’s *post hoc*. Data points on bar graphs indicate spine densities of neuronal dendrites from *n* = 3 mice/treatment group. *: *P* < 0.05, ***: *P* < 0.001. **(D)** Representative immunofluorescence (IF) images (20× magnification) of doublecortin (DCX^+^, green) positive neurons in the suprapyramidal blade of the hippocampal dentate gyrus (DG) from mice administered 3-cycles of VEH, CIS, or CIS+CAF (75 mg/kg). DAPI (gray). Scale bar: 50 μm. **(E)** Quantitative analysis shows that CIS (*n* = 5 brains/treatment) induced significant reductions in DCX^+^ expression in the hippocampal DG, when compared to VEH (*n* = 6 brains/treatment) induced and CIS+CAF (*n* = 5 brains/treatment) induced treated mice. Data points on bar graphs indicate individual mice. One-way ANOVA, Tukey’s *post hoc*. *: *P* < 0.05. Results are reported in mean ± SEM.

Golgi-Cox staining, imaging and dendrite spine reconstruction analysis: we used FD Rapid GolgiStain Kit (FD NeuroTechnologies) to prepare brain sections for Golgi-Cox staining according to the manufacturer’s instructions. Briefly, mice were anesthetized with a ketamine (K;100 mg/kg), xylazine (X;10 mg/kg), and acepromazine (A;10 mg/kg) cocktail (KXA;0.1 ml/10 g body-weight. *i.p.*), followed by decapitation and brain extraction. Brains were then submerged in potassium dichromate, mercuric dichloride and potassium chromate solution in the dark at room temperature (RT) for 3 weeks. Following impregnation, brains were snap-frozen and stored at −80 °C until cryosectioning. Brains were coronally sliced at 100 μm/section, mounted on gelatin-coated slides, stained, and imaged at 100× magnification with a light microscope (Keyence). Secondary branched dendrites within 50 μm from the cell soma of randomly selected neurons in the hippocampal CA1 subregion (Figure 2A) were imaged (Z-pitch: 0.1 μm) and reconstructed for quantitation of dendrite spine densities (Bitplane IMARIS). Fluorescence immunostaining and analysis: briefly, mice were anesthetized with KXA cocktail, followed by cardiac perfusion, first with ice cold PBS, and then 4% paraformaldehyde. Whole brains were extracted and processed for free-floating immunofluorescence staining with primary antibodies specific to Iba-1^+^ microglia, the pro-inflammatory marker CD68, and the neuroblast marker doublecortin (DCX). Primary antibodies were conjugated to appropriate Cyanine Cy3-and-Cy5 secondary antibodies (Jackson ImmunoResearch). Images were acquired with a Thunder Imager 3D microscope (Leica) using Leica LASX software. NIH FIJI was used for analysis of marker expression to calculate density and area. Microglial morphological analysis (Sholl, skeleton and branching) was done with the FIJI Simple Neurite Tracer plugin.

### 2.5 Statistical analysis

Statistics for behavioral and imaging analysis were done with GraphPad Prism 10.2.3 (GraphPad Software, La Jolla, CA, USA). For behavioral and imaging quantitative analysis, we used standard or repeated measures (RM) one, or two-way ANOVA followed by Dunnett’s, Holm-Sidak, or Tukey’s *post-hoc* test for multiple comparisons. In the instance that values were missing, a mixed-effects model was used for ANOVA. Statistical significance was defined as *P* < 0.05. A detailed statistical analysis summary is provided in Supplementary Table 2 in the accompanying Supplementary Information. We used Grubb’s outlier test^1^ to detect statistical outliers from all treatment groups in an unbiased manner. Experiments and data analyses were performed in a blinded fashion.

## 3 Results

### 3.1 Caffeine improves cisplatin-induced deficits in cognition and physicality

#### 3.1.1 Effects of 75 mg/kg caffeine on body-weight recovery, anxiety-like behavior and memory

To investigate the extent that the A_2A_R antagonist caffeine (CAF; 75 mg/kg/day) protects against cisplatin-induced physical and cognitive dysfunction, we provided 30 days of caffeine (1 g/L; Figure 1A) in the drinking water (i.e., free drinking condition) at a dose that improves cognition (Kaster et al., 2015). However, during cisplatin treatment, we ensured hydration in mice by orally gavaging caffeine (75 mg/kg *p.o.*) 1 h prior to cisplatin injection (Figure 1A) and allowing mice to have free access to water in the home cage. There were no significant differences in body weight in our animal cohorts as measured on the day (Day 0) prior to onset of treatment cycles (Supplementary Figure 1A). Weight-loss is common with cisplatin (Moreira-Pais et al., 2018). Notably, caffeine pretreatment in CIS+CAF mice significantly mitigated body-weight deficits when compared to cisplatin alone (Figure 1B). In the Accelerated Rotarod test (Figure 1C), CIS+CAF treated mice displayed comparable fall latencies to vehicle, which were higher than cisplatin-alone. Therefore, our results reflect caffeine’s known improvements to physicality (Aguiar et al., 2020). In the elevated plus maze (EPM), when compared to cisplatin-alone, CIS+CAF treated mice also exhibited increased distance-traveled in the EPM, suggesting caffeine improved cisplatin-induced physical dysfunction (Supplementary Figure 1B). Analysis of time spent in the EPM locations, which is an additional measure of anxiety-like behavior, revealed that CIS+CAF and cisplatin-alone treated mice equally spent significantly more time in the closed-arms and less time in the open-arms when compared to vehicle-alone (Supplementary Figure 1C). However, CIS+CAF treated mice had more center-entries and open-arms entries (Figures 1D,E), suggesting some anxiolytic benefits. Together, our results indicate that caffeine significantly improves cisplatin-induced physical dysfunction. The extent that caffeine confers anxiolytic effects are unclear, with the possibility that the increased entries into exposed EPM locations may be derived from caffeine’s improvements to physicality and less to decreases in anxiety-like behavior. Next, we assessed whether caffeine pretreatment improved spatial learning and memory dysfunctions caused by cisplatin in the Morris water maze (MWM; Supplementary Figure 1D). We found increased swimming speed during Visible Platform Training Day 1 in CIS+CAF treated mice, when compared with cisplatin-alone and vehicle-alone groups, suggesting that caffeine improves physical function (Supplementary Figure 1E). Escape latency analysis revealed that cisplatin-alone (Days 3–4) and CIS+CAF (Days 2–3) treated mice had significantly longer latencies when compared to vehicle-alone controls (Figure 1F). Interestingly, a within-treatment ANOVA analysis detected that CIS+CAF treated mice exhibited a significant decrease in escape latencies when comparing training Day 2-vs.-Day 4 (*P* = 0.038), suggesting task acquisition. However, cisplatin-alone treated mice did not exhibit a similar decrease between Day 2 vs. Day 4 (*P* = 0.893), suggesting impaired task acquisition, as these mice had difficulties in finding the platform (Figure 1F and Supplementary Figure 1E). Vehicle-alone controls showed a significant decrease in escape latency between Day 2-vs.-Day 3 (*P* = 0.003). We then assessed spatial memory during the MWM Probe Test (Day 5) where the platform was removed and performance during the first trial (NE-release point; Supplementary Figure 1G) was examined (Vorhees and Williams, 2006). Analysis of latencies to the first platform cross from the NE-release point showed that mice treated with cisplatin-alone had significantly longer delays in comparison to CIS+CAF and vehicle-alone treated mice (Figure 1G). Measurement of the frequency of platform crosses (Supplementary Figure 1H) and SE target quadrant crosses (Supplementary Figure 1I) during the Probe test detected a significant decrease in crossings by cisplatin-alone treated mice when compared to vehicle-alone. Interestingly, CIS+CAF treated mice displayed a trend toward more frequent platform crosses and SE quadrant crosses in comparison to cisplatin-alone treated mice, although the differences were not statistically significant (Supplementary Figures 1H,I). We did not detect differences in % time spent in the SE target quadrant (Supplementary Figure 1J). Notably, both CIS+CAF and vehicle-alone treated mice had significantly faster swim speeds when compared to cisplatin-alone during the Probe test (Supplementary Figure 1K), again indicating improved physicality by caffeine. During visible platform Day 6, vehicle-alone treated mice had significantly faster escape latencies in comparison to cisplatin-alone and CIS+CAF treated mice (Supplementary Figure 1L). Despite the analysis showing that CIS+CAF treated mice had on average faster escape latencies than cisplatin-alone treated mice, there were no statistical differences detected between the two groups. Nonetheless, there were no swim speed differences detected between the treatment groups when the platform was visible (Supplementary Figure 1M), suggesting that poor task performance in the MWM was not due to physical or visual impairments. Taken together, our results suggest that while caffeine may offer some modicum of protection against cisplatin-induced spatial memory deficits, this interpretation must be taken with caution as there exists the possibility that the improved performance by caffeine in the MWM may stem from caffeine’s enhancements to physicality.

### 3.2 Caffeine attenuates cisplatin-induced dendrite spine density impairments in the hippocampus

Dendrite spine density is a cellular hallmark of cognitive function, while spine density deficits are associated with cognitive decline (Sikora et al., 2021). Our recent study demonstrated that A_2A_R inhibition by istradefylline prevents stunting of dendrite length in hippocampal post-mitotic adult-born neurons caused by cisplatin (Oliveros et al., 2022). We thus examined if caffeine (75 mg/kg) offered neuroprotection against cisplatin-induced spine density deficits in CA1 hippocampal pyramidal neurons (Figure 2A). 3D reconstruction of Golgi-Cox stained neurons revealed that caffeine pretreatment in CIS+CAF mice had comparable dendrite spine densities to vehicle-alone, with both being significantly higher than cisplatin-alone, suggesting that caffeine attenuated cisplatin-induced dendrite spine deficits (Figures 2B,C).

### 3.3 Caffeine attenuates cisplatin-induced impairments to hippocampal neurogenesis

We recently reported that cisplatin detrimentally impairs expression of the neural stem cell (NSC) neuroblast marker doublecortin (DCX), in the hippocampal dentate gyrus (DG). Importantly, these negative effects were prevented by the A_2A_R antagonist istradefylline (Oliveros et al., 2022). Remarkably, our immunofluorescence analysis revealed that in comparison to cisplatin-alone, caffeine at 75 mg/kg (Figures 2D,E) and 37.5 mg/kg (Supplementary Figures 2A,B) in CIS+CAF treated mice had DCX levels comparable to vehicle-alone and caffeine-alone, indicating that caffeine conferred significant protection against cisplatin-induced neurogenic impairments.

### 3.4 Cisplatin induces a primed, activated state in hippocampal microglia that is attenuated by caffeine

**FIGURE 3:**
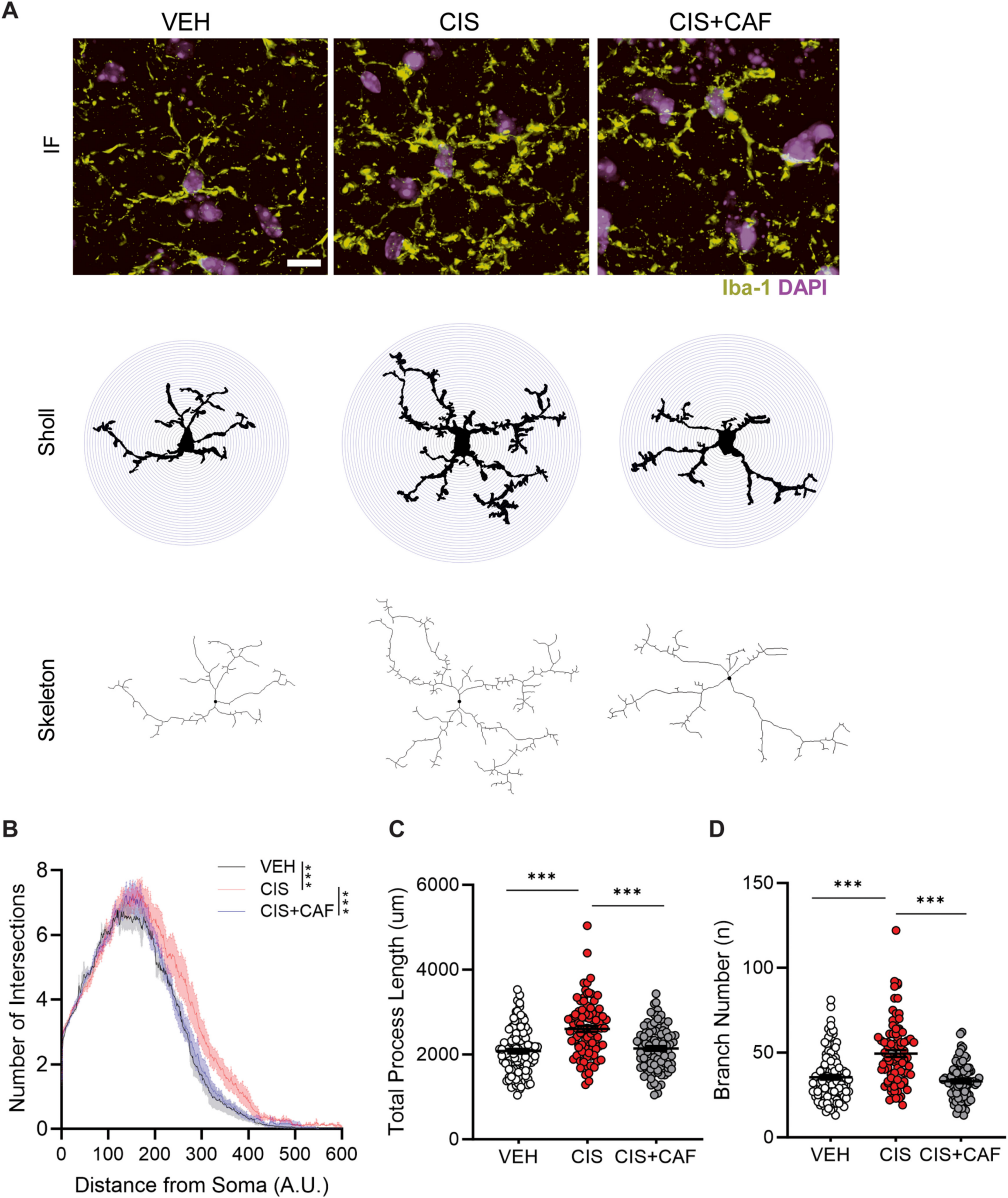
Caffeine attenuates cisplatin-induced microglial priming in the CA1 subregion of the hippocampus. **(A)** Representative immunofluorescence (IF) images (63× magnification) of Iba-1^+^DAPI^+^ colocalized microglia in the CA1 subregion of the hippocampus from mice that were administered vehicle (VEH), 3-cycles of cisplatin (CIS; 2.3 mg/kg, *i.p.*) or mice that were pretreated with caffeine (75 mg/kg, *p.o.*) in combination with cisplatin (CIS+CAF). Representative IF images were converted to binary mask and skeletonized for Sholl analysis, quantitation of total process length, and branch number. Scale bar: 5 μm. Iba-1 (green), DAPI (blue). **(B)** Sholl analysis indicates that microglia in VEH (*n* = 110 microglia) and CIS+CAF (*n* = 90 microglia) treated brains exhibited significantly shorter processes when compared to CIS-alone (*n* = 89 microglia). CIS-alone treated microglia were morphologically more complex, as evidenced by a higher number of concentric circles distal from the cell soma that intersected processes. Arbitrary Units (A.U.). Microglia analyzed are derived from *n* = 4 brains/treatment group. Linear regression: Slope *P* = 0.062, Elevation/Intercept = *P* < 0.001; Two-way ANOVA, Tukey’s *post hoc*. ***: *P* < 0.001. **(C)** Skeletonization analysis identified that CIS-alone (*n* = 89) treated microglia displayed significant increases in total process length in comparison to VEH (*n* = 110 microglia) and CIS+CAF (*n* = 90 microglia) treated brains. Microglia analyzed are derived from *n* = 4 brains/treatment group. One-way ANOVA, Tukey’s *post hoc*. ***: *P* < 0.001. **(D)** Skeletonization analysis identified that CIS-alone (*n* = 89) treated microglia displayed significant increases in branching complexity in comparison to VEH (*n* = 110 microglia) and CIS+CAF (*n* = 90 microglia) treated brains. The data points in each bar graph represent individual microglia derived from *n* = 4 brains/treatment group. One-way ANOVA, Tukey’s *post hoc*. ***: *P* < 0.001. Results are reported in mean ± SEM.

**FIGURE 4:**
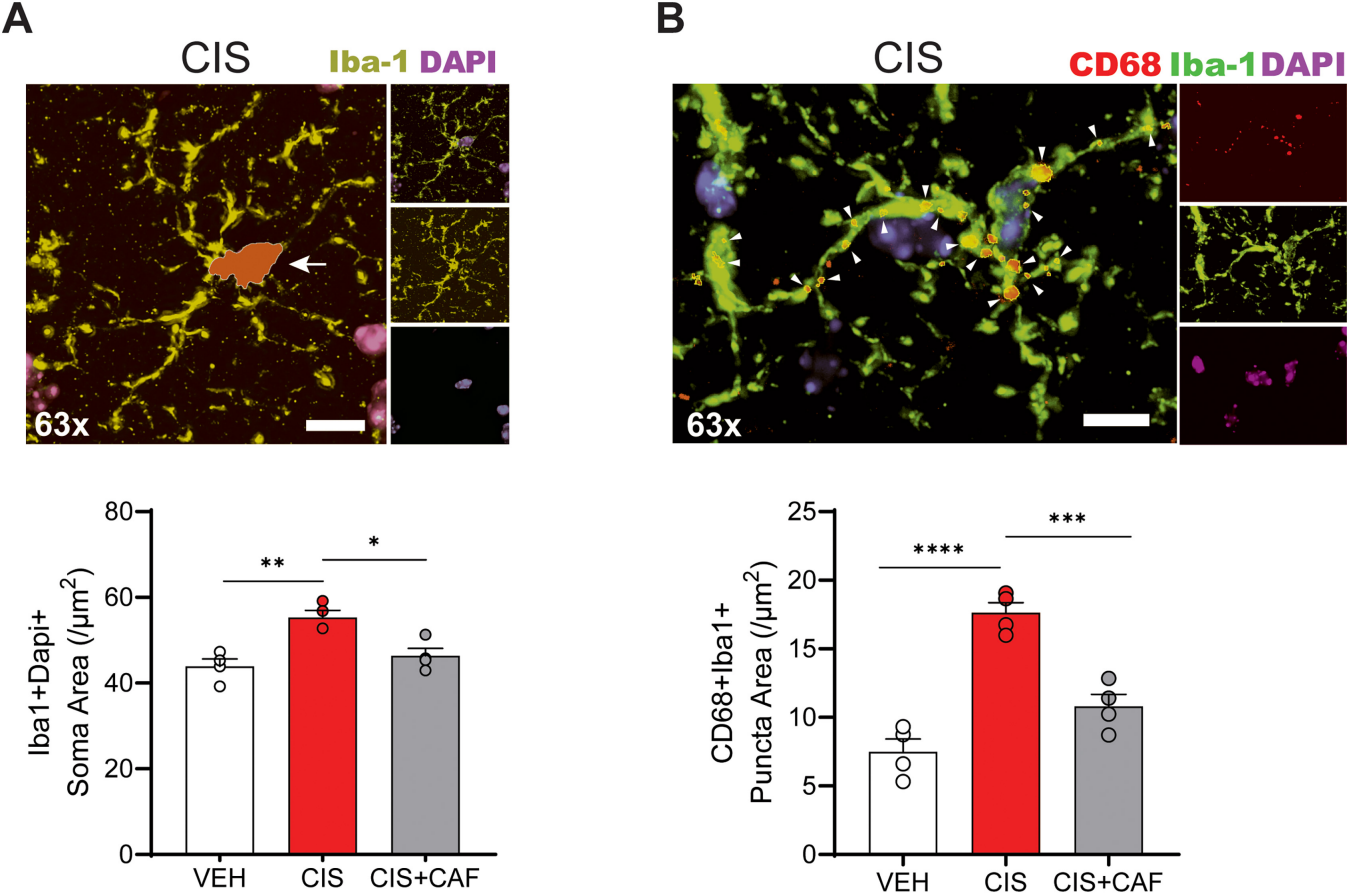
Caffeine attenuates cisplatin-induced neuroinflammatory activation of microglia in the CA1 subregion of the hippocampus. **(A)** Representative immunofluorescence (IF) image (63× magnification) of a Iba-1^+^DAPI^+^ microglia from cisplatin (CIS; 2.3 mg/kg, *i.p.*) treated brain that was overlayed with a traced cell soma outline (red) in the hippocampal CA1 of mice. Quantitative analysis of Iba-1^+^DAPI^+^ hippocampal microglia from brains of mice administered 3-cycles of vehicle (VEH), CIS, or caffeine (75 mg/kg, *p.o.*) in combination with cisplatin (CIS+CAF), shows that CIS treatment significantly increases cell soma area, when compared to VEH and CIS+CAF treated mice. Iba-1 (green), DAPI (blue). Scale bar: 5 μm. CA, Cornu *Ammonis*. Data points in bar graphs represent cell soma measurement. VEH: *n* = 110–167 microglia measured from 2 to 3 regions of interest (ROI)/section, 4 sections/brain and *n* = 4 brains/treatment. CIS: *n* = 98–114 microglia measured in 2–3 ROI/section, 3–4 sections/brain and *n* = 4 brains/treatment. CIS+CAF: *n* = 152–193 microglia measured from 3 ROI/section, 4 sections/brain and *n* = 4 brains/treatment. One-way ANOVA, Tukey’s *post hoc*. *: *P* < 0.05, **: *P* < 0.01. **(B)** Representative IF images depicting colocalized expression of the inflammatory marker CD68 in Iba-1^+^ microglia in the hippocampal CA1 subregion of a CIS treated brain. Quantitative analysis of CD68^+^ puncta expression in Iba-1^+^ microglia from mice given 3-cycles of VEH, CIS, or CIS+CAF (75 mg/kg, *p.o.*). Indicate that CIS increases CD68^+^ area (μm^2^) when compared to VEH, whereas caffeine pretreatment attenuates increased CD68^+^ expression in CIS+CAF treated microglia. CD68 (red), Iba-1 (green), DAPI (blue). Scale bar: 5 μm. Data points in bar graph represent CD68^+^ area. VEH: CD68^+^ area (μm^2^) measurements from *n* = 21–26 Iba-1^+^ microglia in 2 ROI/section, 3 sections/brain and *n* = 4 brains/treatment. CIS: CD68^+^ area measured of *n* = 21–23 Iba-1^+^ microglia in 2 ROI/section, 3 sections/brain and *n* = 4 brains/treatment. CIS+CAF: CD68^+^ area measured of *n* = 35–43 Iba-1^+^ microglia from 1 to 3 ROI/section, 3–4 sections/brain and *n* = 4 brains/treatment. One-way ANOVA, Tukey’s *post hoc*. ***: *P* < 0.001. Results are reported in mean ± SEM. ****P < 0.00001 derived from a one-way ANOVA post-hoc analysis.

Microglial priming and surveillance have been identified as an intermediary pro-inflammatory response in preclinical models of brain injury, stress vulnerability, and accelerated aging (Morrison and Filosa, 2013; Hui et al., 2018; Takahashi et al., 2021). We therefore asked whether cisplatin induces primed microglial morphology. In comparison to vehicle-alone and CIS+CAF treated mice, Sholl analysis from skeletonized microglia of cisplatin-alone treated hippocampus show significantly more intersections (Figures 3A,B), as well as increased process length and branching complexity (Figures 3C,D), suggesting that caffeine (75 mg/kg) in CIS+CAF treated mice prevented cisplatin-induced microglial priming. When compared to vehicle-alone and CIS+CAF treated mice (Figure 4A and Supplementary Figure 3C), cisplatin-alone microglia displayed larger cell soma area, suggesting caffeine attenuated activation (Pomilio et al., 2016). Importantly, we found that the microglial activation marker CD68 (Hoogland et al., 2015; Corujo-Ramirez et al., 2020) was higher in cisplatin-alone Iba1^+^ microglia, when compared to vehicle-alone and CIS+CAF treated mice (Figure 4B and Supplementary Figure 3D). Interestingly, caffeine in CIS+CAF treated mice did not reverse cisplatin-increased microglial density in the hippocampus (Supplementary Figures 3A,B). Our results suggest that A_2A_R antagonism by caffeine attenuates cisplatin-induced CD68 expression and morphological characteristics resembling microglial activation.

### 3.5 Effects of 37.5 mg/kg caffeine against cisplatin-induced CICI

To investigate if a lower concentration of caffeine (37.5 mg/kg p.o.; approximately two 20-oz cups of coffee) attenuated cognitive, cellular, and physical dysfunction caused by cisplatin, we shortened caffeine (1 g/L) pre-exposure to 5 days and orally gavaged caffeine upon onset of cisplatin treatment cycles (Supplementary Figure 4A). Although there were no significant differences in body weight in our animal cohorts as measured on the day (Day 0) prior to onset of treatment cycles, in comparison to cisplatin-alone, caffeine in CIS+CAF treated mice promoted faster body weight recoveries as these mice reached comparable weights to vehicle-alone and caffeine-alone groups faster (Supplementary Figures 4B,C). Although more delayed, cisplatin-alone treated mice eventually reached (Day 67) comparable body-weights to vehicle-alone and caffeine-alone groups (Supplementary Figures 4B,C). These results support caffeine as an ergogenic option against physical frailty exerted by cisplatin. In terms of memory function, we found that in comparison to cisplatin-alone, caffeine pretreatment in CIS+CAF mice improved Y-maze working memory that was comparable to vehicle-and-caffeine alone controls (Supplementary Figures 4D,E). Analysis of locomotor function did not detect a significant difference in distance traveled between CIS+CAF and cisplatin-alone treated mice, suggesting that caffeine improvements to cognition in this test could not be solely attributed to enhanced motor function (Supplementary Figure 4F). Analysis of anxiety-like behavior in the EPM showed that in comparison to cisplatin-alone, caffeine (37.5 mg/kg) pretreatment in CIS+CAF treated mice increased open-arm entries comparably to caffeine-alone and vehicle-alone cohorts (Supplementary Figure 5A). Notably, vehicle-alone, caffeine-alone, and CIS+CAF treated mice took significantly less time than cisplatin-alone treated mice to enter the unprotected center location (Supplementary Figure 5B). Analysis of center-entries revealed that compared to cisplatin-alone, CIS+CAF treated mice had higher entry frequencies, albeit these differences were not statistically significant. We did detect significantly more center-entries in vehicle-alone and caffeine-alone treated mice (Supplementary Figure 5C). Although both vehicle-alone and caffeine-alone treated mice exhibited higher distance traveled, we did not detect significant differences in locomotion between CIS+CAF and cisplatin-alone treated mice (Supplementary Figure 5D). In terms of time spent in the closed vs. the unprotected EPM locations, we did not detect significant differences of time spent in the closed or open-arms of the maze between cisplatin-alone and by 37.5 mg/kg caffeine in CIS+CAF treated mice. Both vehicle-alone and caffeine-alone treated mice spent significantly less time in the closed-arms and more time in the open-arms of the EPM when compared to cisplatin-alone and CIS+CAF treated mice (Supplementary Figure 5E). Taken together, both 37.5 and 75 mg/kg caffeine in CIS+CAF treated mice did not increase time spent in the exposed/unprotected maze locations (e.g., center, open-arms), suggesting that chronic caffeine in our studies did produce aberrant anxiogenesis. Instead, caffeine in CIS+CAF treated mice increased open-arm entries and center-entries, suggesting the potentiality that caffeine may have benefits against cisplatin induced deficits in anxiety-like behavior. Despite this, caffeine did not improve cisplatin-induced decreases in time spent in exposed maze locations, the traditional anxiety measure in this test, while the enhancements in open-arm entries and center-entries in the EPM may stem from caffeine’s ability to bestow benefits against cisplatin-potentiated impairments to physical function. Consequently, our EPM results should be interpreted with caution.

## 4 Discussion

Preclinical and clinical studies report physical frailty and neuroinflammation that negatively affects QoL in cancer survivors (Belcher et al., 2022; Carroll et al., 2022). CICI is hypothesized to negatively affect cognition through neuroinflammatory elevation of cytokines and microglial activation (Gibson et al., 2019; Subramaniam et al., 2022; Kim et al., 2024). Although early reports did not find microglial activation in the brain following cisplatin exposure (Chiu et al., 2017; George et al., 2021), our results support mounting evidence indicating that cisplatin chemotherapy activates microglia (Kim et al., 2023, 2024). Notably, our studies are the first to elucidate that cisplatin promotes microglial priming typified by elevations in the activation marker CD68, in conjunction with a larger cell soma, highly complex branching and extended processes in the hippocampus. As a critically important brain region for learning, memory and emotive behavior, the vulnerability of the hippocampus to neurotoxicity by chemotherapy cannot be underscored (Anacker and Hen, 2017; Nevins et al., 2023). This suggests that cisplatin potentiates a primed hyper-surveillant microglial phenotype, which remarkably, was attenuated by the A_2A_R antagonist caffeine. In agreement, caffeine has been shown to attenuate microglial activation, process extension and branching following hypoxic-ischemic conditions (Steger et al., 2014; Boia et al., 2017; Di Martino et al., 2020; Yang et al., 2022). Additionally, the A_2A_R antagonist SCH58261 prevents microglial process extension/ramification caused by glucocorticoid-induced stress (Caetano et al., 2017), while deletion of the adenosine generating enzymes (CD39, CD73) inhibit microglial process extension/ramification, further implicating adenosine in regulation of microglial process dynamics (Matyash et al., 2017). Indeed, decreased neuronal activity by anesthesia, neurotoxic brain injury (Nimmerjahn et al., 2005; Morrison and Filosa, 2013; Liu et al., 2019; Umpierre et al., 2020; Haruwaka et al., 2024), or psychological depression (Maras et al., 2022; He et al., 2024), promotes microglial process extension and hyper-ramification, transiently shifting toward primed hyper-surveillance. Consequently, we posit that microglia may be detecting cisplatin-induced synaptic impairments, prompting process extension, hyper-ramification, and increased branching complexity, indicative of primed hypersurveillance. Additional studies are warranted to determine how chemotherapy negatively affects microglial process dynamics and whether A_2A_R functionally controls these phenotypes in the context of CICI. We previously showed that the FDA approved A_2A_R antagonist istradefylline mitigates body-weight loss caused by cisplatin (Oliveros et al., 2022). Our present studies support these findings since A_2A_R antagonism by caffeine similarly mitigated body-weight loss caused by cisplatin. We propose that caffeine’s inhibitory effects on neuronal A_2A_R may be a mechanism, given that caffeine does not improve ergogenic function in mice with forebrain deletion of A_2A_R (Aguiar et al., 2020). In addition to the cognitive protective effects against cisplatin which we detected in the Y-maze, the physical improvement by caffeine was reflected by improved motor function in the accelerated Rotarod, the EPM and the Morris water maze. This suggests that elevated levels of caffeine during chemotherapy could bestow physical benefits beyond chemotherapy cessation. For example, mice pretreated with caffeine plus cisplatin exhibited more frequent entries and faster latencies to enter exposed/unprotected EPM maze locations. While this may suggest some corrective benefits to cisplatin-induced anxiety, we advise caution with this interpretation, since caffeine did not improve cisplatin-induced decreases in the time spent in the exposed/unprotected arms, which is the traditional anxiety measure for this assay. Similarly, in the Morris water maze, in spite of the ability for caffeine ability to improve physical performance in this test, the protective benefits against cisplatin-induced spatial memory dysfunction were only very modest. Consequently, additional research is required before asserting with certainty that caffeine offers spatial memory protection against cisplatin. Our present study shows that chronic caffeine exposure confers neuroprotective effects to adult-born hippocampal neurogenesis and dendrite spine densities in CA1 post-mitotic neurons. This complements our recent report describing how specific A_2A_R antagonism by istradefylline attenuates cisplatin-induced impairments to DCX expression in the hippocampal DG, and neuronal morphology in the hippocampal CA1 (Oliveros et al., 2022). Remarkably, these detriments to DCX expression were long lasting, up to 42 days following the final cisplatin injection, thus reflecting clinical studies that describe CICI in patients years post chemotherapy treatment (Koppelmans et al., 2012; Nguyen et al., 2013; Gregorowitsch et al., 2019).

### 4.1 Study limitations

Although our findings are in agreement with studies indicating caffeine is a putative cognitive enhancer (Cognato et al., 2010; Pandolfo et al., 2013; Onaolapo and Onaolapo, 2015; Machado et al., 2017), our study is not without limitations. First, although microglial activation by cisplatin suggests neuroinflammation, we did not measure levels of proinflammatory cytokines in the brain. Therefore, other than microglial activation, we cannot conclude with certainty that caffeine attenuated other neuroinflammatory sequelae caused by cisplatin. Furthermore, the studies in our current manuscript do not use an A_2A_R-specific inhibitor or a model of A_2A_R knockout/knockdown. To clarify the extent that microglial priming by cisplatin in CICI is a process that is controlled by caffeine’s non-specific inhibition of A_2A_R, will necessitate additional mechanistic investigations. Second, since we only used female mice, our studies did not address potential sex differences in our chemobrain model. Our rationale for selecting female mice was that recent studies suggest that chemotherapy treated female survivors – both adult and pediatric – exhibit higher incidences of symptomatic and objective neurological adverse events, including deficits in mood, memory, sleep, and strategic planning (Unger et al., 2022; Colliva et al., 2024). Sex dependent vulnerability to CICI have been shown in preclinical rodent studies (Zamorano et al., 2023; Fowler et al., 2025), and interestingly, chemotherapy sensitivity may vary with the female estrous cycle. Tumors are more sensitive to genotoxic treatment during oestrus (high estrogen/low progesterone) than during diestrus (low estrogen/high progesterone), suggesting that hormonal status may influence vulnerability to CICI (Boue et al., 2024; Bornes et al., 2025). Future studies are needed to examine how cisplatin chemotherapy differentially affects cognitive function by sex, and whether caffeine confers similar neuroprotective benefits against cisplatin-induced microglial primed hypersurveillance and cognitive impairment in males. A third limitation is that we did not measure liquid intake or assess circulating caffeine levels in blood during our experiments. Therefore, we were unable to ascertain the extent that caffeine in the drinking water (i.e., free drinking condition) or caffeine via oral gavage differentially affected the physiological state and behavioral performance of our cohorts. Consequently, we were unable to mechanistically determine how A_2A_R inhibition by caffeine promotes neurocellular protection against cisplatin-induced chemobrain. However, experiments done by B.B. Fredholm’s group demonstrated that orally administered caffeine (∼70 mg/kg) for 14 days conferred neuroprotection and reduced mortality against NMDA potentiated clonic seizures (Georgiev et al., 1993), while 100 mg/kg caffeine transcriptionally activates genes (NGF1A-B, c-Fos, c-Jun) essential for neuronal differentiation, synaptic integrity and cognitive function (Svenningsson et al., 1995a,b). Although speculative, it is possible that chronic caffeine in our studies could promote transcriptional activity of the aforementioned genes to afford neuroprotection against cisplatin. Surprisingly, studies with caffeine within the dose range used in our studies report inhibition of pro-apoptotic and DNA damage response factors, as well as protection against lethal doses of radiation induced weight loss (George et al., 1999; Kim et al., 2003, 2005). Additional preclinical studies are needed to mechanistically determine the extent that A_2A_R inhibition by caffeine at lower, more clinically feasible doses can potentially exert therapeutic efficacy against CICI. An additional caveat is that the doses of caffeine used in our studies have been reported to produce tachycardia and anxiogenesis, as well as impair cognitive function and sociability (Baldwin et al., 1989; Kaplan et al., 1997; Hinton et al., 2019; Mahdi et al., 2019). It is unclear if inclusion of a 75 mg/kg caffeine-alone control group in our studies would have revealed either robust physical enhancements, or anxiogenesis (e.g., in the EPM), thus limiting our interpretation of the results. Nonetheless, our caffeine-alone control at 37.5 mg/kg did not increase time spent in the closed maze locations above control levels, suggesting that chronic caffeine exposure did not result in aberrant anxiogenesis. Finally, we would like to note that although our chronic caffeine strategy (e.g., 75 mg/kg) maintained higher levels of caffeine than the daily average human consumption, the safety profile of caffeine is evident, as we did not observe caffeine-induced mortality in any of our cohorts. Indeed, we were well below caffeine’s lethal dose (187–200 mg/kg) reported for mice (Seale et al., 1984; Bonati et al., 1985; George et al., 1999). Adherence to chronic caffeine intake against CICI would not be difficult given its widespread use and well-established safety profile. For example, several recent clinical studies report that long term intake of elevated caffeine levels are associated with lower risk of dementia and higher cognitive improvement (Zhang et al., 2026), reduced risk of breast cancer (Oh et al., 2015), and reduced risk and mortality from colorectal cancer (Sinha et al., 2012; Mackintosh et al., 2020). Interestingly, caffeine, in combination with exercise, inhibited neuroinflammation and protected cognition in a rat model of sleep deprivation (Vastegani et al., 2025). However, additional studies are necessary to determine individualized strategies (e.g., dose and duration of caffeine intake) that would balance clinically effective caffeine intake that does not negatively affect sleep or exacerbate chemotherapy induced insomnia (Bean et al., 2022; Maccora et al., 2022). In conclusion, the findings in our study indicates for the first time that A_2A_R antagonism by caffeine may represent a neuroprotective role in CICI.

## Data Availability

The original contributions presented in this study are included in this article/Supplementary material, further inquiries can be directed to the corresponding author.

## Footnotes

1
https://www.graphpad.com/quickcalcs/grubbs1/

## Supplementary material

Supplementary Table 1 Treatment groups in experiment 1 (75mg/kg caffeine) and experiment 2 (37.5 mg/kg caffeine).

Supplementary Table 2 Summary of statistical analysis.
