# Supplementary material for: Caffeine attenuates cisplatin induced microglial reactivity and cognitive dysfunction
Source: Front Mol Neurosci. 2026 May 13;19:1772430. doi: 10.3389/fnmol.2026.1772430 (PMC13212328; doi:10.3389/fnmol.2026.1772430)
Supplement: Supplementary Table 1 — Treatment groups in experiment 1 (75mg/kg caffeine) and experiment 2 (37.5 mg/kg caffeine). [file Supplementary_file_1.docx]

**Caffeine attenuates cisplatin-induced microglial reactivity and cognitive dysfunction**

**Alfredo Oliveros^1, 2, 3, *^, Ivan Loncar^1^, Marwan Mostafa^1^, Michael Poleschuk^1^, Ana M. Corujo^3^, Bo Qin^4^, Juhyun Song^5^** **, Max A. Tischfield^6^, Mi-Hyeon Jang^1, 3^**

^1^Department of Neurosurgery, Robert Wood Johnson Medical School, Rutgers University, Piscataway NJ 08554, USA.

^2^Department of Biological Sciences, The State University of New York at Buffalo, Buffalo NY 14260, USA.

^3^Department of Neurological Surgery, Mayo Clinic College of Medicine, Rochester MN 55905, USA.

^4^Section of Cancer Epidemiology and Health Outcomes, Rutgers Cancer Institute, New Brunswick, NJ 08901, USA.

^5^Department of Anatomy, Chonnam National University Medical School, Hwasun, 58128, Republic of Korea.

^6^Department of Cell Biology and Neuroscience, School of Arts and Sciences, Rutgers University, Piscataway NJ 08554, USA.

* Correspondence to Alfredo Oliveros at alfredoo@buffalo.edu

1. Supplementary Material and Information

1.1. Mouse husbandry

All experiments were performed on 3-4 month old female C57BL/6J mice (Jackson Laboratory, Bar Harbor ME, USA). All mice were housed in standard ventilated cages under a 12-hour light/dark cycle (lights on at 0600 h and off at 1800 h) with water and food provided *ad libitum* in the home cage. Mice were randomly allocated to each treatment group prior to the onset of treatments and animal identification was done via marks on the tail with a non-toxic black marker. Tail markings were repeated as necessary to reliably identify animals throughout treatment cycles, during behavior testing, and until experimental endpoints. For behavior experiments, mice were habituated for at least 30 min to the behavior testing room and mice underwent testing starting at 1300 h. Water and food was provided *ad libitum* in the home cage. Mouse experiments were conducted in accordance with National Institutes of Health guidance on the care and use of laboratory animals. All procedures were approved by the Institutional Animal Care and Use Committee (IACUC) of Mayo Clinic and the Rutgers University IACUC.

1.2. Drug administration

1.2.1. Experiment 1: Effects of 75 mg/kg caffeine against cisplatin administration

We have previously shown that cisplatin increases adenosine A_2A_R levels in the hippocampus which exerts cognitive dysfunction (Oliveros et al., 2022). Therefore, we hypothesized that chronic pre-exposure to the non-specific adenosine A_2A_R antagonist caffeine, would confer neuroprotection against cisplatin-induced chemobrain.

*Cisplatin treatment regimen:* In our studies, one treatment cycle consists of a once daily cisplatin injection (2.3 mg/kg *i.p.*/day, dissolved in 0.9% saline, delivered at 0.1mL/10 g body weight; Fresenius Kabi, Lake Zurich IL, USA) or vehicle injection (0.9 % saline), administered for 5 consecutive days, followed by 5 days of rest from injections, and this treatment schedule was repeated for 3-cycles (Yoo et al., 2021;Oliveros et al., 2022). This dosing regimen of cisplatin has been previously demonstrated to pathologically accumulate in dorsal root ganglia neurons to exert peripheral neuropathies (Gill and Windebank, 1998;Ta et al., 2009), as well as potentiate neurocellular and cognitive dysfunction akin to clinical cognitive impairments (Le and Hanna, 2018;Szturz et al., 2019). Although cisplatin’s half-life in the mouse circulatory compartment has been reported to be from 15-33 min (Daley-Yates and McBrien, 1984;Gondal et al., 1993), this chemotherapy readily distributes and persists in malignant and normal tissues beyond 24 h (Breglio et al., 2017;Perse, 2021), including the hippocampus and pre-frontal cortex (Nakagawa et al., 1996;Yoo et al., 2021).

*Caffeine regimen prior to cisplatin treatment cycles:* Caffeine, when provided in the drinking water (Ardais et al., 2014;Hinton et al., 2019), exerts psychoactive properties that improve cognition and protect against maladaptive stress responses (Duarte et al., 2009;Cognato et al., 2010;Kaster et al., 2015). Given the persistence of cisplatin in normal and malignant tissues, we used a forced choice paradigm to administer water-alone or caffeine (1g/L; Cat# C0750; MilliporeSigma, St. Louis MO, USA) in a water bottle for 30 days to elevate caffeine levels prior to onset of cisplatin administration (Fig. 1A and Supplementary Table 1). During this 30 day period, the water delivery system in the ventilated rack housing system was disconnected.

*Caffeine regimen during cisplatin treatment cycles:* Given that caffeine is a known diuretic (Seal et al., 2017), and cisplatin can detrimentally reduce water intake (Perse, 2021), instead of caffeine in the drinking water (free drinking paradigm), we opted to provide caffeine through oral gavage to ensure hydration during chemotherapy treatment (Cat# 18061-22; Fine Science Tools, Foster City CA, USA). Hence, mice had *ad libitum* access to water via the ventilated home cage rack system (Fig. 1A and Supplementary Table 1). Mice were given 75 mg/kg/day (3.75 mg/mL, *p.o.;* approximately four 20-oz cups of coffee) of caffeine via oral gavage 1 h prior to cisplatin injections to determine the extent of maintaining elevated caffeine levels against cisplatin accumulation. Vehicle-alone or cisplatin-alone treated mice were given water via oral gavage 1 h prior to their respective injections (Fig. 1A and Supplementary Table 1). During the 5-day rest from cisplatin or vehicle injections, mice continued to receive caffeine or water via oral gavage (Fig. 1A). All oral gavage administrations were delivered at a dose volume of 0.2mL/10 g body weight. Importantly, our selected dose of caffeine is similar to doses shown to be protective against lethal doses of gamma radiation (George et al., 1999), and our selected dose is also well below the lethal dose for caffeine (187 - 200 mg/kg) reported for mice (Seale et al., 1984;Bonati et al., 1985;George et al., 1999).

*Caffeine regimen following cisplatin treatment cycles:* Following the final cisplatin or vehicle treatment injection, we ceased to provide caffeine or water via oral gavage (due to the stress of this procedure) and instead provided access to caffeine (1g/L) or water via the home cage water bottle. To ensure hydration, all mice were also allowed water access via the ventilated cage rack system.

1.2.2. Experiment 2: Effects of 37.5 mg/kg caffeine against cisplatin administration

*Caffeine regimen prior to cisplatin treatment cycles:* As depicted in Supplementary Fig. 3A and Supplementary Table 1, we modified our caffeine administration approach and reduced caffeine (1g/L) pre-exposure to 5-days prior to onset of cisplatin or vehicle treatment. Similar to Experiment 1, water delivery via the ventilated rack housing system was disconnected during this 5-day caffeine pre-exposure period.

*Caffeine regimen during cisplatin treatment cycles:* In this experiment, we administered caffeine (37.5 mg/kg *p.o.*) via oral gavage 1 h prior to cisplatin injections to determine if a lower concentration of caffeine was efficacious in providing neuroprotection against cisplatin-induced cognitive and cellular deficits (Supplementary Fig. 3A and Supplementary Table 1). Similar to Experiment 1, to maintain hydration in mice upon onset of cisplatin (2.3 mg/kg/day, *i.p.*) or vehicle (0.9 % saline) treatment cycles (Supplementary Fig. 3A and Supplementary Table 1), mice were allowed unrestricted *ad libitum* access to water delivered in the home cage via the ventilated rack housing system. Mice that were injected with cisplatin-alone or vehicle-alone received water via oral gavage 1 h prior to injections. A separate group of mice received water or caffeine pre-exposure (1 g/L; caffeine-alone group) for 5-days and were orally gavaged with water or 37.5 mg/kg *p.o.* of caffeine 1 h prior to vehicle (0.9% saline) injections (Supplementary Fig. 3A and Supplementary Table 1). All oral gavage administrations were delivered at a dose volume of 0.2mL/10 g body weight.

In contrast to Experiment 1, upon completion of cisplatin or vehicle treatment cycles, caffeine treated mice were not allowed to have caffeine in water-bottles in the home cage. During recovery, all mice had *ad-libitum* access to water from the ventilated rack housing system.

1. 3. Body weight measurements

Physiological frailty is a common phenotype experienced by adult and pediatric cancer survivors administered chemotherapy (Ness et al., 2013;Demaria et al., 2017;Wang et al., 2021), including survivors treated with cisplatin (Moreira-Pais et al., 2018). Our previous study indicated that specific A_2A_R antagonism by the FDA approved drug istradefylline significantly improved body weight recovery following cisplatin chemotherapy (Oliveros et al., 2022). Our selection of mice at 3-4 months of age ensured that our experimental cohorts would have sufficiently robust body weight prior to cisplatin treatment. All mice were healthy at the start of our experiments, and we used body weight as a determinant of health in our mouse cohorts to detect a frailty-like phenotype resulting from chemotherapy. Body weight measurements were recorded during treatment cycles, behavior testing, and the recovery period following treatment cycles as indicated for each experiment.

1.4. Behavioral analysis

Mice were transferred from colony housing to the behavior testing suite and allowed acclimation to the behavior room for 1-hour before onset of testing to minimize stress during behavior testing days. During behavior testing all mice were provided water via water bottles in the home cage. At the end daily experimental testing, mice were returned to their home cage and transferred back to colony housing. For behavior testing, a monochrome camera mounted to the ceiling of the testing suite was used for video recording of behavior experiments. Tracking analysis was performed with EthoVision-XT 14 software (Noldus Information Technology, Leesburg, VA, USA). Where appropriate, testing chambers were sanitized with 70% ethanol between trials or with soap and water between experiments.

*1.4.1. Elevated Plus Maze*

Given inherent proclivity of the rodent to cautiously explore unfamiliar spaces, the elevated plus maze (EPM; ENV-560A, Med-Associates St. Albans VT, USA) examines anxiety-like behavior in the rodent by quantifying time spent in open, unprotected sections of the mazee relative to closed sections of the maze, in a 5 min period (Walf and Frye, 2007;Oliveros et al., 2022). The EPM is elevated 50 cm above the floor. Mice are released at the open center location (L 6 cm x W 6 cm) between the open and closed arms of similar dimensions (L 34.9 cm x W 6 cm), and allowed to freely explore the maze (Supplementary Fig. 1A). The closed arms are lined with a 26 cm high opaque black wall. Distance traveled and time spent in each maze location (center area, open and closed arms), latency to 1^st^ center entry, and entry frequency into the center and open arms were recorded and analyzed (Noldus).

*1.4.2. Morris Water Maze*

Morris water maze (MWM) performance requires intact hippocampal function, and the MWM test is widely to study spatial learning and memory in rodents (Vorhees and Williams, 2006). Relying on navigational learning of the location of spatial cues around a blue circular tank (120 cm diameter and 90 cm deep; ENV594M-B, Med-Associates) to find and escape onto a concealed, submerged platform (10.2 cm diameter; ENV-596M, Med-Associates). Concealment of the submerged platform (1.25 cm below water surface) was achieved by mixing non-fat dry milk in water. Four visible spatial cues of different colors (red, orange, white, green), shaped like a star, square, circle and triangle, were placed at North (N), South (S), East (E), and West (W) cardinal coordinate locations, thus dividing the tank into four quadrants (Supplementary Fig. 1D). To measure learning during MWM training, mice were released from an equidistant location between each cardinal coordinate point (i.e., NE, SE, SW, NW release points) where swim latencies and trajectories to escape onto the hidden platform were analyzed (Noldus). The test was performed across 6 consecutive days consisting of visible platform days (Day 1 and Day 6). Training to find the submerged, concealed platform was done on Days 2 – 4, and a memory probe test day was performed on Day 5, where the platform was removed (Supplementary Fig. 1D). For every MWM test day, a mouse was first released from the NE release point, followed by every mouse in the cage for that treatment group before sequentially moving on to the next release point (i.e., NE🡪SE🡪SW🡪NW). Following completion of a trial, mice were removed from the platform and placed in a drying cage containing dry paper towels until the next release trial. A heat pad was placed underneath the drying cage to keep mice warm. *Visible Platform Day 1*: For each release trial, mice were allowed a maximum of 2 min to find the platform, visibly located the SE quadrant (signaled by an orange flagpole protruded 8 cm above the water’s surface). If a subject found the platform in less than 2 min, mice had to remain on the platform for at least 5 s before video tracking stopped, ending the trial. If a mouse failed to locate and escape onto the platform, they were then guided to swim to the platform. For this day only, regardless of whether a subject located and escaped onto the platform, all mice were allowed to remain on the platform for 30 s to habituate mice to the platform. *Training Days 2–4*: The flag was removed, and mice had to rely on spatial cues to learn to navigate and escape onto the submerged platform. During each test day, mice were sequentially released from each release point and allowed 2 min to find the platform. If mice found the platform in less than 2 min, the trial ended, like Day 1. If mice were unable to locate and escape onto the platform within 2 min, they were manually guided to swim to the platform and allowed to remain there for an additional 30 seconds. For data analysis of MWM performance during training days and visible platform days, the escape latencies from each release point were averaged for each animal per day. *Memory Probe Test Day 5*: To specifically probe spatial memory retention, the hidden platform was removed and the first trial where mice were released from the NE point was analyzed for the number of SE quadrant crosses, latency to the first platform cross, frequency of target platform crosses, latency to the first SE quadrant cross, % time spent in the SE quadrant and swim speed were measured. *Visible Platform Day 6*: Conditions for this day was similar as in Days 1 – 4, except the platform was visibly located the SE quadrant (signaled by the orange flagpole). *Data analysis*: Swim speed, latency to escape onto the platform was measured for Day1 – 4, and Day 6. Water temperature was maintained between 25 – 27 ± 1 °C across testing days.

*1.4.3. Y- Maze*

Analysis of continuous spontaneous alternation in the Y-maze has been previously utilized to investigate working memory by relying on whether mice can successfully recall which arm was previously explored (Hughes, 2004;Albani et al., 2014). Y-maze performance has been investigated in the context of cognitive improvements by caffeine (Cognato et al., 2010;Pandolfo et al., 2013;Onaolapo and Onaolapo, 2015), and conversely, cognitive disruptions by 2-cycles of cisplatin (Chiu et al., 2017;Chiu et al., 2018;Ma et al., 2018). To determine if caffeine could abrogate working memory deficits from 3-cycles of cisplatin treatment, we assessed continuous spontaneous alternation in the Y-maze, which possessed three identical, equidistant arms (40 cm long x 16 cm high x 11.5 cm wide) separated from each other by a 120° angle (Supplementary Fig. 3D). Arms were designated A, B, and C and all mice were released from Arm A, facing the experimenter and opposite the center of the maze. Operationally, a correct alternation meant that mice entered arms sequentially, such that entries into arms C🡪A🡪B or arms B🡪A🡪C or arms A🡪C🡪B were scored as 3 correct alternations. Accordingly, the maximum number of alternations are derived from the total number of arm entries minus 2, and percentage alternation was as previously described (Onaolapo and Onaolapo, 2015). $\left( \frac{Correct Alternations}{Maximum Alternations} \right)x 100.$ Mice were allowed to freely explore the maze for 5-min, where entries into arms were video tracked and distance traveled as well as alternations, were calculated (Noldus).

*1.4.4. Accelerated Rotarod*

We evaluated the effects of 3-cycles of cisplatin administration on Accelerated Rotarod performance and investigated whether caffeine (75 mg/kg p.o.) provided motor-learning improvements against the frailty-like motor dysfunction caused by cisplatin administration (Callizot et al., 2008;Hussien and Yousef, 2022). Mice underwent Rotarod testing across three trials separated by 30 min between each trial, using a standard Rotarod treadmill (Ugo Basile, Verese, Italy) which was programmed to gradually accelerate from a baseline level of 2 rpm, and terminate at a maximal speed of 40 rpm over a 300 second period (Oliveros et al., 2017). Latency to fall from the ridged, rotating beam was used as our measure of motor-learning and coordination.

1.5. Brain Preparation and Imaging Analysis

1.5.1. Golgi-Cox Staining

To prepare brains for Golgi-Cox staining, we used FD Rapid GolgiStain Kit (Cat# PK401A, FD NeuroTechnologies, Inc. Columbia MD, USA) according to the manufacturer’s instructions and as previously described (Du, 2019). Following administration of 3-cycles of cisplatin-alone, caffeine (75 mg/kg, *i.p.*; Fig. 1A) in combination with cisplatin (CIS+CAF), or vehicle-alone, and rest from behavior testing, mice were anesthetized with a ketamine (K; 100 mg/kg), xylazine (X; 10 mg/kg), and acepromazine (A; 10 mg/kg) cocktail (KXA; 0.1ml/10g body weight. *i.p.*) as previously described (Yoo et al., 2021;Oliveros et al., 2022). Briefly, mice were decapitated and whole brains were swiftly extracted, rinsed in ultra-pure water to remove blood, and submerged in potassium dichromate, mercuric dichloride and potassium chromate impregnation solution (FD NeuroTechnologies). Brains were then stored in the dark at room temperature (RT) for 3 weeks. Following the 3-week impregnation period, brains were rapidly frozen using pre-cooled (~70-80 °C) isopentane (Cat # M0167, TCI America, Portland OR, USA) and stored at -80 °C until cryosectioning (Leica CM1850; Leica Biosystems, Wetzlar, Germany). Brains were coronally sliced at 100 µm/section, from anterior to posterior at ~ - 23 °C and mounted on gelatin coated slides (Cat # PO101, FD NeuroTechnologies). Sections were then stained and subsequently dehydrated with increasing concentrations of ethanol (50%, 75%, 95% and 100%), followed by xylene immersion. Golgi-Cox stained sections were then cover-slipped using Permount solution (Cat # SP15-500, ThermoFisher Sci, Waltham MA, USA), and stored in the dark at RT prior to imaging.

1.5.2. Microscopy and Analysis of 3D Reconstructed Dendrite Spines

Three separate regions of interest (ROI) were selected for imaging of Golgi-Cox stained neurons in the Stratum Oriens layer of the hippocampal CA1 subregion (Fig. 4A). Imaging was performed with a Keyence BZ-X microscope (Keyence Corp., Itasca IL, USA) using a 100x oil immersion objective. Within each ROI, randomly selected individual neurons were identified, taking care to capture the cell soma, as well as primary, secondary, and tertiary basal dendrites within Z-stacks (Z-pitch at 0.1 µm). To reconstruct and quantify dendrite spines from Z-stack images of individual neurons, we utilized Bitplane Imaris 3D (v 8.02, Oxford Instruments, Oxfordshire UK), selecting secondary dendrites that were within 50 µm from the cell soma for analysis. Starting at the base of the secondary branch where it diverged from the primary branch, tracing approximately 25 µm and spine counts were automatically standardized utilizing a 1/10um rubric. To set dendrite spine detection seed point thresholds, we averaged measurements of 3-4 random spine heads from each reconstructed image. Dendrite traces and analysis was performed from 1-6 dendrites per neuron and at least 3 neurons across each ROI (Fig. 4), totaling n = 5-11 individual neurons/treatment, and n = 3 brains per treatment group.

1.5.3. Brain Preparation for Immunostaining

Following administration of treatment cycles and behavior testing in Experiment 1 (75 mg/kg, *i.p.*; Fig. 1A), and similarly following behavior testing and recovery in Experiment 2 (37.5 mg/kg, Supplementary Fig. 3A), mice were anesthetized with KXA (0.1ml/10g body weight. *i.p.*) followed by cardiac perfusion with ice cold 1x PBS and then perfused with 4% paraformaldehyde. Whole brains were then carefully extracted and processed for histological analysis as previously described (Hussaini et al., 2013;Oliveros et al., 2022). Briefly, entire brains were sectioned in coronal slices 40 µm thick from anterior to posterior, in serial order, and processed for free floating immunostaining with primary antibodies specific to the microglia marker Ionized Ca^2+^ Binding Adaptor protein-1 (goat Iba-1, 1:500; Cat#: ab289876, Abcam, Cambridge UK), the inflammatory marker CD68 (rat anti-mouse, 1:500; Cat#: MCA1957, BioRad, Hercules CA, USA). We implemented antigen retrieval (Hussaini et al., 2013) for visualization of the immature neuron marker doublecortin (DCX, rabbit, 1:250; Cat#4604S, Cell Signaling Technologies, Danvers MA, USA). Primary antibodies were conjugated to appropriate Cyanine Cy3 and Cy5 secondary antibodies (Jackson ImmunoResearch, West Grove PA, USA) and DAPI was used as a nuclei counter stain (Cat#: H2000; Vector Labs, Newark CA, USA).

1.5.4. Microscopy and Image Analysis

For image acquisition of microglia located in the hippocampal CA1, we utilized a Thunder Imager 3D Tissue fluorescence microscope (Leica, Wetzlar, Germany) using immersion oil at 63x magnification and a Z-step size of 2 µm. For analysis of microglia numbers, we used the Cell Counter Plugin in NIH ImageJ’s FIJI, and only Iba-1^+^DAPI^+^ colocalized microglia with processes that could be traced back to the cell soma were counted. For measurement of microglia cell soma area (µm^2^), we used the free-hand tracing feature and ROI manager in FIJI and all Iba-1^+^DAPI^+^ colocalized microglia within each image was selected for analysis. For area measurement of CD68^+^ expression in Iba-1^+^ microglia, we used the magic wand tool to trace puncta and the ROI manager in FIJI. Scale bars were derived from calculated distances in images acquired with Leica LASX software. For density, cell soma area measurements, and CD68 puncta area analysis, at least n = 3 microglia were selected from an individual imaged ROI, across three distinct ROIs per hippocampal CA1 brain section (similar to the areas selected for analysis of Golgi-Cox stained neurons, as depicted in Fig. 4A), and at least n = 3 hippocampal sections (from anterior to posterior), per mouse brain.

For morphological evaluations of microglial processes, only Iba-1^+^DAPI^+^ colocalized microglia were selected for semi-automated tracing with the FIJI SNT plugin toolbox (v4.2.1), followed by binary conversion (sigma 3.0 pixel, weight 0.6), skeletonization, and Sholl analysis for identifying the number of intersections distal from the cell soma, total process length, and branch number (Morrison and Filosa, 2013;Ferreira et al., 2014;Young and Morrison, 2018;Arshadi et al., 2021). As a criterion for analysis, at least 1─5 Iba-1^+^DAPI^+^ colocalized microglia were selected in an individual ROI, across three distinct ROIs per hippocampal CA1 brain section, and at least 3 sections (from anterior to posterior) per mouse brain. Only Iba-1^+^DAPI^+^ colocalized microglia with processes that could be traced back to the cell soma were used for semi-automated tracing. For Sholl analysis intersections plots, we utilized a start radius of 1, and an arbitrary step size of 0 to accurately capture morphological changes as concentric circles emanated from the cell soma. Following skeletonization analysis, total process length for each microglia was derived from multiplying the average process length by the detected number of branches.

For image acquisition of the hippocampal dentate gyrus (DG) we used 20x magnification, a Z-step size of 2 µm, at least n = 2 hippocampal sections (from anterior to posterior), per mouse brain, and measured the subgranular zone (SGZ) area with Leica LASX software. For quantitative analysis of DCX^+^ cells in the hippocampal SGZ, we used the Cell Counter Plugin in NIH ImageJ’s FIJI.

1.6. Statistical Analysis

All statistical analyses were executed using GraphPad Prism 10.2.3 (GraphPad Software, La Jolla, CA, USA) as shown in Supplementary Table 2 depicting our statistical analysis. For behavioral and imaging quantitative analysis, we used standard or repeated measures (RM) one or two-way ANOVA followed by Dunnett’s, Holm-Sidak, or Tukey’s *post-hoc* test for multiple comparisons, as appropriate for each experiment. In the instance that values were missing, a mixed-effects model was used for ANOVA. For Sholl intersection analysis, we performed a simple linear regression analysis, followed by a standard two-way ANOVA, in conjunction with Tukey’s *post-hoc* testing to compare intercepts of concentric intersections across treatment groups. Statistical significance was defined as *P* < 0.05 (*, *P* < 0.05; **, *P* < 0.01; ***, *P* < 0.001). For results that were *P* > 0.05, they were defined as statistically not significant (n.s.). We used Grubb’s outlier test (https://www.graphpad.com/quickcalcs/grubbs1/) to detect statistical outliers, which were removed equally from all treatment groups in an unbiased manner. Experiments and data analyses were performed in a blinded fashion.

| Supplementary Table 1. Treatment Groups in Experiment 1 (75mg/kg caffeine) and Experiment 2 (37.5 mg/kg caffeine) | | | | | |
| --- | --- | --- | --- | --- | --- |
| **Experiment 1** |  |  |  |  |  |
| **Treatment group** | **VCS water before onset of treatment cycles?** | **Treatment in home cage bottle** | **Injections (*i.p.*) during cycles** | **Gavage treatment (*p.o.*)** | **VCS water during intervention?** |
| Vehicle-alone | None for preceding 30 days | -Water for 30 days prior to injections  -Water during behavior | 0.9% saline | Water | Injections: Yes  Behavior: Yes |
| Cisplatin-alone | None for preceding 30 days | -Water for 30 days prior to injections  -Water during behavior | 2.3 mg/kg cisplatin in 0.9% saline | Water | Injections: Yes  Behavior: Yes |
| Cisplatin + Caffeine | None for preceding 30 days | -Caffeine (1 g/L) for 30 days prior to injections  -Caffeine (1 g/L) during behavior | 2.3 mg/kg cisplatin in 0.9% saline | 75 mg/kg p.o. (3.75 mg/mL) Caffeine in water | Injections: Yes  Behavior: Yes |
|  |  |  |  |  |  |
| **Experiment 2** |  |  |  |  |  |
| **Treatment Name** | **VCS Water Before onset of treatment cycles?** | **Treatment in home cage bottle** | **Injections (*i.p.*) during cycles** | **Gavage treatment** | **VCS water during intervention?** |
| Vehicle-alone | None for preceding 5 days | -Water for 5 days prior to injections | 0.9% saline | Water | Injections: Yes  Behavior: Yes |
| Cisplatin-alone | None for preceding 5 days | Water for 5 days prior to injections | 2.3 mg/kg cisplatin in 0.9% saline | Water | Injections: Yes  Behavior: Yes |
| Cisplatin + Caffeine | None for preceding 5 days | 1 g/L caffeine for 5 days prior to injections | 2.3 mg/kg cisplatin in 0.9% saline | 37.5 mg/kg p.o. (1.875 mg/mL) Caffeine in water | Injections: Yes  Behavior: Yes |
| Caffeine-alone | None for preceding 5 days | 1 g/L caffeine for 5 days prior to injections | 0.9% saline | 1.875 mg/mL Caffeine in water | Injections: Yes  Behavior: Yes |

Ventilated Cage System = VCS


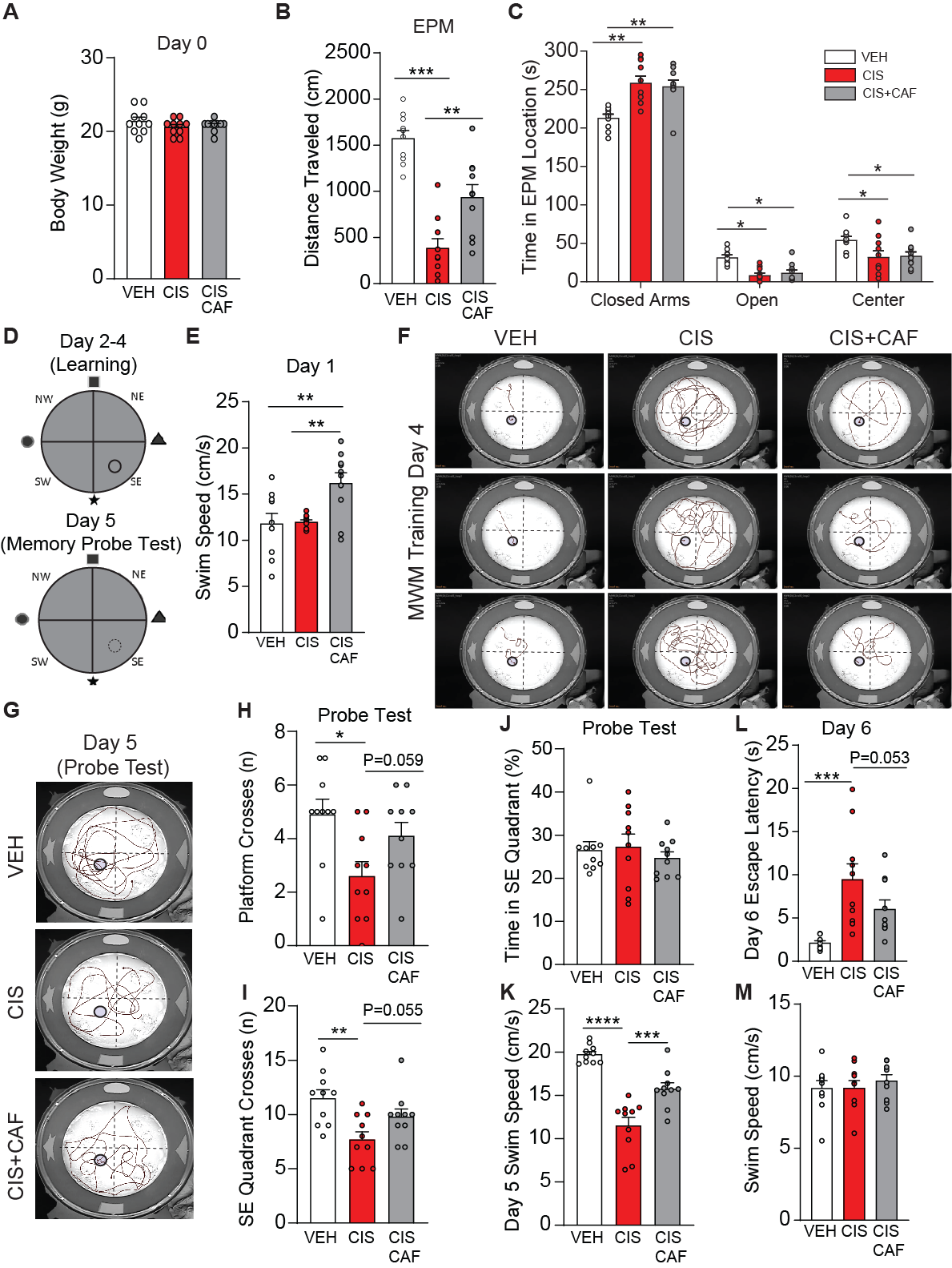


**Supplementary Fig. 1. Caffeine improves physical performance in the elevated plus maze and Morris water maze.** (**A**) Analysis of body weight on the day preceding onset of treatment cycles does not show a significant difference between the treatments. 1-way ANOVA, Dunnett’s *post-hoc*. (**B**) Compared to vehicle-alone (VEH) treated mice and mice pretreated with caffeine followed by cisplatin (CIS+CAF), cisplatin-alone (CIS) treated mice displayed significant decreases in locomotor distance during the elevated plus maze (EPM). (**C**) Time spent in EPM location shows that both CIS+CAF and CIS treated mice spent more time in the closed-arms and less time in the center location and open-arms when compared to VEH. (**D**) Diagram shows the Morris water maze (MWM) circular tank divided into four quadrants signaled by a spatial cues shaped as a square (North), triangle (East), star (South), circle (West) at each cardinal point and each release point (NE, SE, SW, NW) as well as the location of the submerged platform (circle in SE quadrant) on Training Days 2-4 and Day 5 (Memory Probe Test where the hidden platform (dashed circle in SE quadrant) examines spatial memory recall. (**E**) Compared to VEH and CIS-alone treated mice, CAF treated mice displayed faster swimming speed during visible platform training Day 1. (**F**) Representative traces depicting trials from VEH (n=3 different mice), CIS (n=3 different mice) and CIS+CAF (n=3 different mice) released from the SW point and their trajectories to find the submerged platform in the SE quadrant. (**G**) Memory Probe test (Day 5): Representative traces of VEH, CIS and CIS+CAF treated mice showing trajectories and crosses at the former location of the platform in the SE quadrant. (**H**) During the first release trial (NE release point) of the MWM memory probe test (Day 5), CIS treated mice had significantly less platform crosses when compared to VEH treated mice, however we did not detect significant differences in the frequency of platform crosses between CIS and CIS+CAF treated mice. (**I**) Memory Probe test (Day 5): Quantitative analysis of SE quadrant crosses from mice released from the NE quadrant shows that VEH treated mice exhibited significantly more platform crossings (located in SE quadrant) in comparison to CIS and CIS+CAF treated mice, without significant differences detected in platform crossings between CIS and CIS+CAF treated mice. (**J**) Memory Probe test (Day 5): Analysis of % time spent in the SE quadrant did not detect differences between VEH, CIS and CIS+CAF treated mice. (**K**) Memory Probe test (Day 5): Analysis of swim speed from mice released from the NE quadrant shows that VEH and CIS+CAF treated mice displayed significantly improved swimming performance when compared to CIS-alone treated mice. (**L**) Visible Platform Day 6: Analysis of swim speed when the escape platform was visible (SE quadrant) did not detect differences between VEH, CIS and CIS+CAF treated mice, suggesting that deficits in MWM performance was not due to detriments in physical ability.(**M**) Visible Platform Day 6: Analysis of escape latency when the escape platform was visible (SE quadrant) shows that VEH treated mice had significantly faster escape latencies in comparison to CIS treated mice. There were no significant differences in escape latencies between CIS and CIS+CAF treated mice. (**B**, **C**, **E**): 1-way ANOVA, Tukey’s *post-hoc*. n = 10 mice/treatment group. (**C**): 2-way ANOVA, Tukey’s *post-hoc*. n = 10 mice/treatment group. (**H**, **I**, **J**, **K**, **L**, **M**): 1-way ANOVA, Holm-Sidak’s *post-hoc*. n = 10 mice/treatment group. * P ≤ 0.05, ** P ≤ 0.01, *** P ≤ 0.001, **** P ≤ 0.0001, ns: not significant. Data points in bar graphs represent individual animals. Results are reported in mean ± SEM.


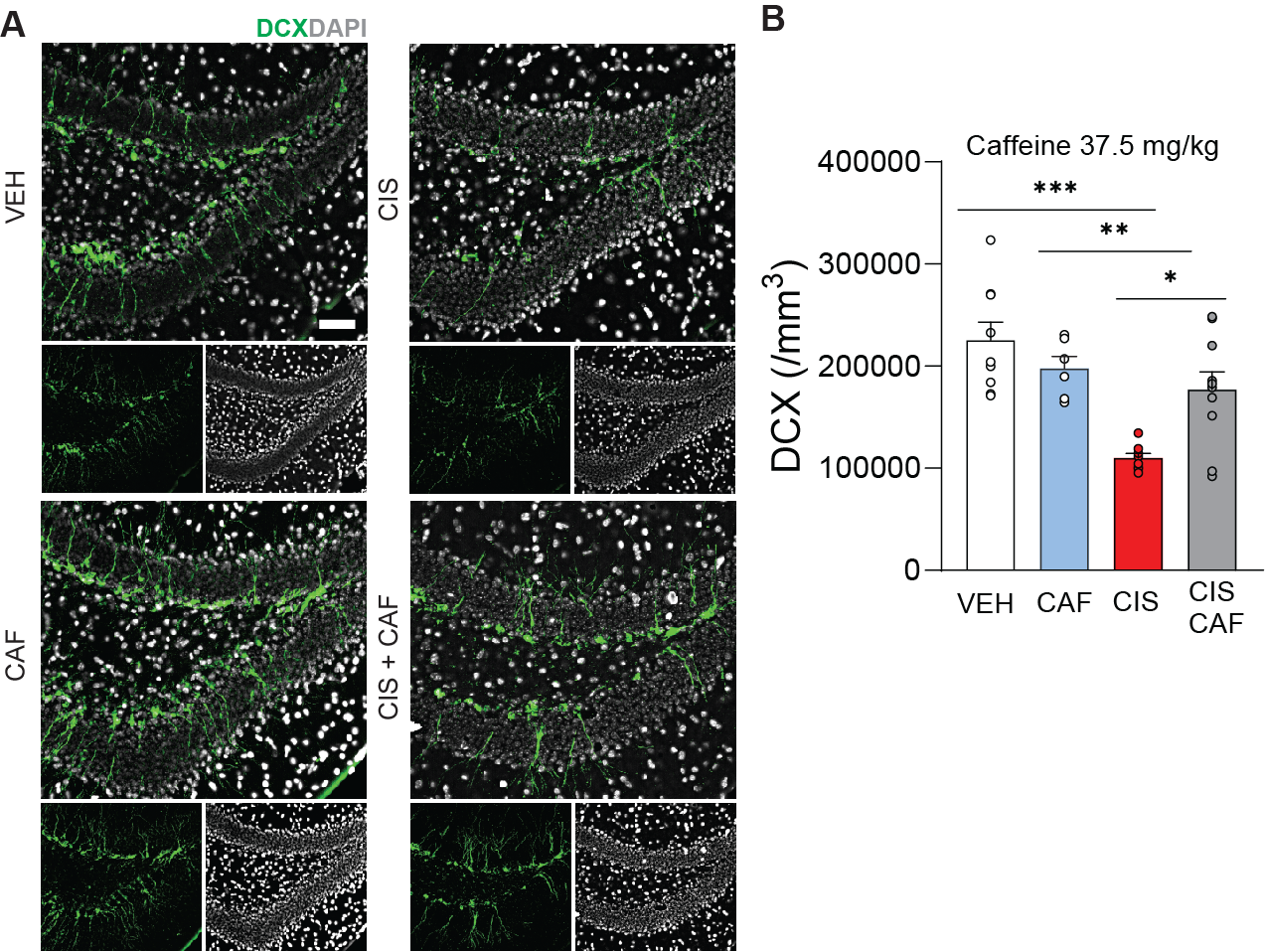


**Supplementary Fig. 2. Caffeine (37.5 mg/kg) attenuates cisplatin-induced neurogenesis impairments in the hippocampal dentate gyrus.** (**A**) Representative immunofluorescence (IF) images (20 x magnification) showing doublecortin (DCX^+^, green) positive neurons in the crest, suprapyramidal and infrapyramidal blades of the hippocampal dentate gyrus (DG) from mice administered 3-cycles of vehicle (VEH), cisplatin (CIS; 2.3 mg/kg, *i.p.*), caffeine-alone (CAF; 75 mg/kg, *p.o.*) or mice that were pretreated with caffeine in combination with cisplatin (CIS+CAF). DAPI (grey). Scale bar: 50 µm. (**B**) Quantitative analysis of shows that when compared to VEH and CAF treated mice, CIS treated mice had significant reductions in DCX^+^ expression in the hippocampal DG, an effect that was attenuated by caffeine in CIS+CAF treated mice. n = 6-10 brains/treatment. One-way ANOVA, Tukey’s *post-hoc*. *: *P* < 0.05, **: *P* < 0.01, ***: *P* < 0.001. Results are reported in mean ± SEM.


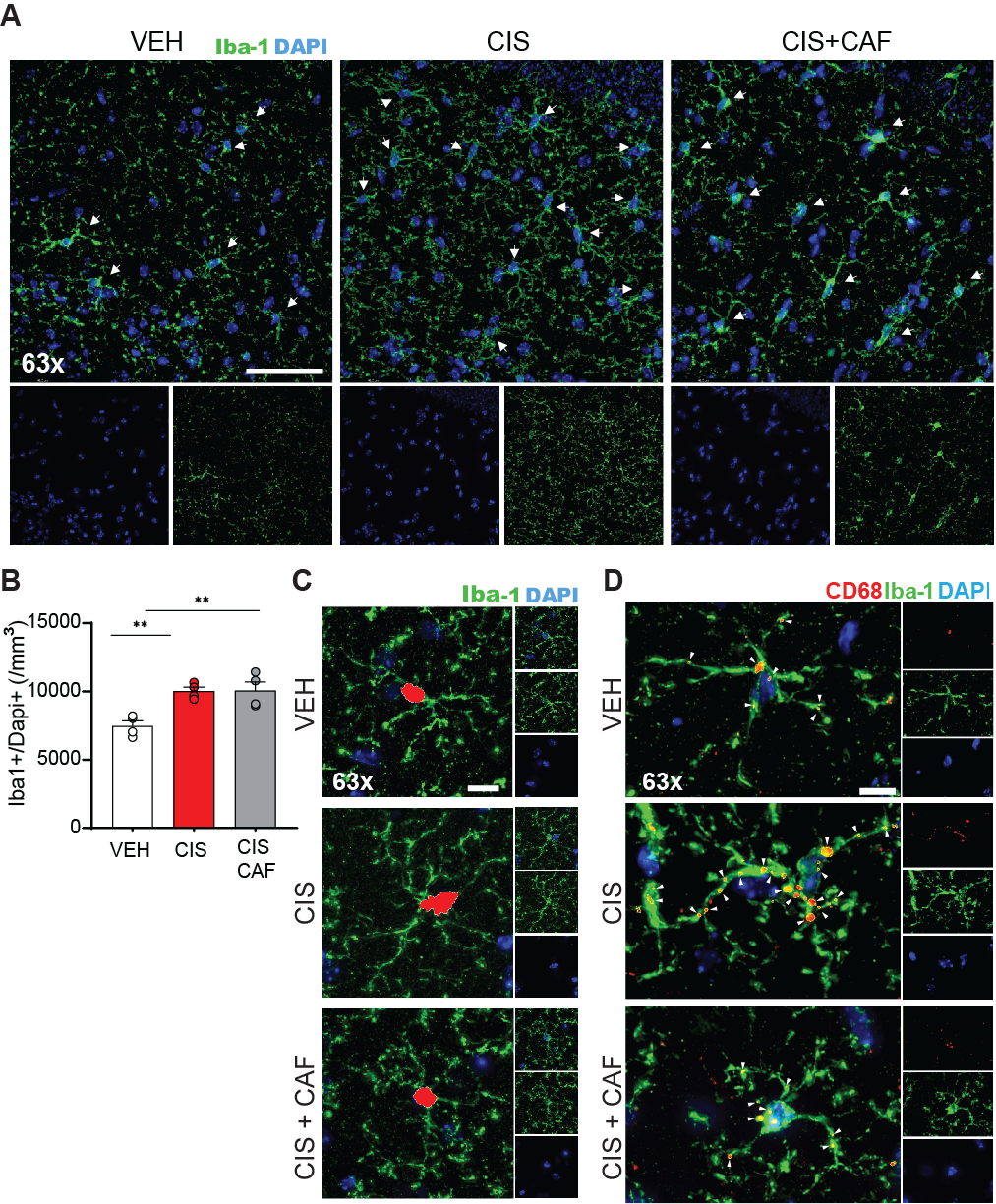


**Supplementary Fig. 3. Cisplatin increases microglial density in the CA1 subregion of the hippocampus.** (**A**) Representative immunofluorescence images (63 x magnification) of Iba-1^+^ microglia (white arrows) in the CA1 subregion of the hippocampus, and (**B**) quantitative analysis shows increased numbers of microglia in mice administered 3-cycles of cisplatin (CIS; 2.3 mg/kg, *i.p.*) or in mice that were pretreated with caffeine (75 mg/kg, *p.o.*) in combination with cisplatin (CIS+CAF), when compared to vehicle (VEH) treated mice. Scale bar (red): 50 µm. Iba-1 (green), DAPI (blue). Data points in each bar graph represent the number of calculated microglia from individual mouse brains analyzed in each treatment group that are derived from a total of n = 370 microglia counted in 1-3 ROI's per section, 3-4 sections/brain and n = 4 brains/treatment. For CIS treated mice, the number of calculated microglia are derived from n = 446 microglia counted in 2-3 ROI's per section, 3-4 sections/brain and 4 brains/treatment. For CIS+CAF treated mice, the number of calculated microglia are derived from n = 462 microglia counted in 1-3 ROI's per section, 4 sections/brain and 4 brains/treatment. One-way ANOVA, Tukey’s *post-hoc*. **: *P* < 0.01. Results are reported in mean ± SEM. (**C**) Representative immunofluorescence (IF) image (63 x magnification) of a Iba-1^+^DAPI^+^ microglia from VEH, CIS and CIS+CAF treated brain that was overlayed with a traced cell soma outline (red) in the hippocampal CA1 of mice. Scale bar: 5 µm. (**D**) Representative IF images (63 x magnification) depicting colocalized expression of the inflammatory marker CD68 in Iba-1^+^ microglia in the hippocampal CA1 subregion of a CIS treated brain. CD68 (red), Iba-1 (green), DAPI (blue). Scale bar: 5 µm.


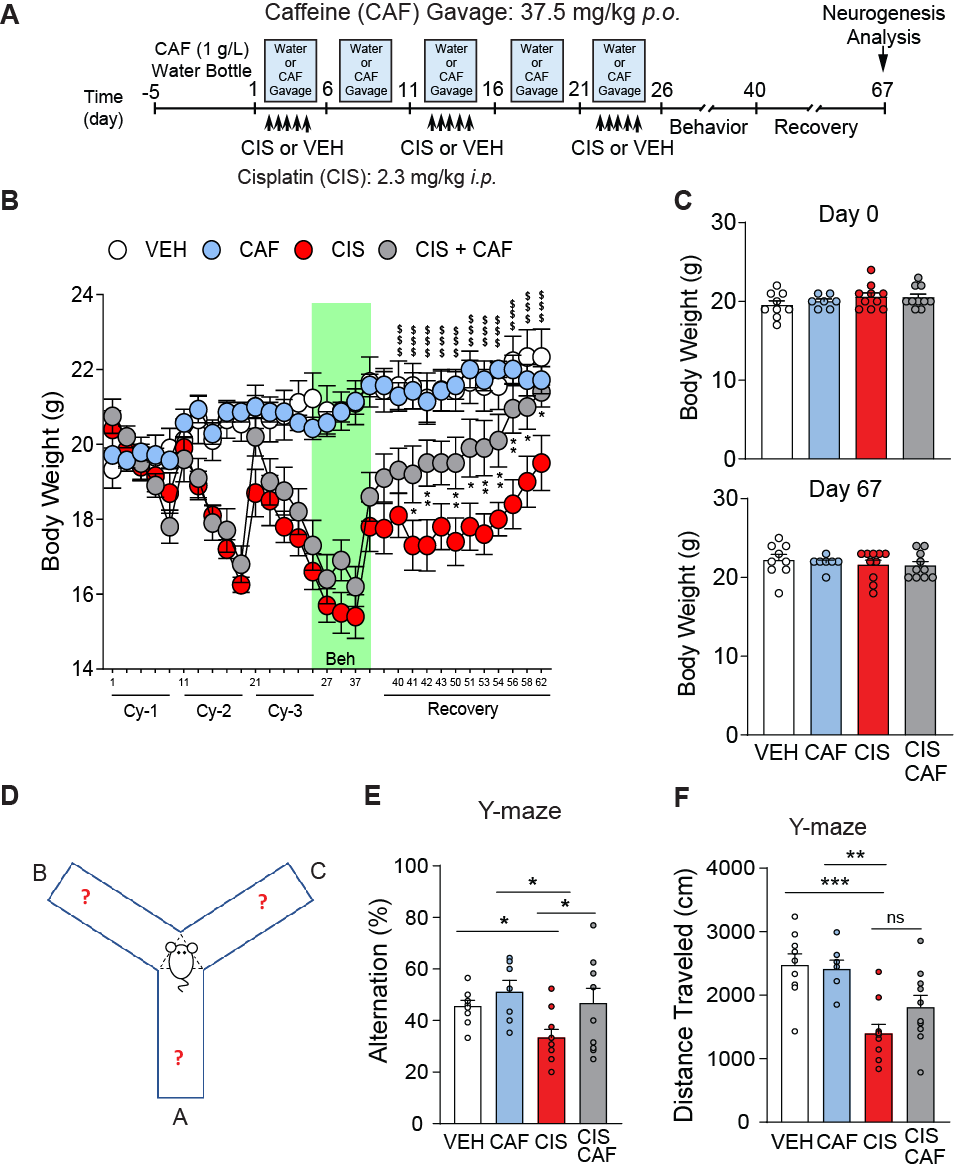


**Supplementary Fig. 4. Caffeine (37.5 mg/kg) exposure hastens physical recovery from cisplatin chemotherapy, improves physical function in the EPM and cisplatin-induced working memory deficits in the Y-maze.** (**A**) Timeline and experimental design of caffeine pretreatment (1 g/L in the drinking water), followed by oral gavage of water or caffeine-alone (CAF; 37.5 mg/kg, *p.o.*), or oral gavage of caffeine plus injections of cisplatin (CIS+CAF), oral gavage of water plus cisplatin-alone injections (CIS; 2.3 mg/kg *i.p.*) or oral gavage of water plus vehicle-alone (VEH) injections. Timeline also shows follow up behavior assays, recovery from treatment cycles and neurogenesis analysis. Upward arrows indicate daily consecutive CIS injections. (**B**) Body-weight analysis of VEH, CIS, and CIS+CAF, and CAF treated mice across 3-cycles of treatment, during behavior testing and recovery from CIS treatment. CIS+CAF treated mice had body-weights that approached comparable VEH and CAF control levels faster, when compared to CIS treated mice. (**C**) Analysis of body weight on the day preceding onset of treatment cycles (Day 0, upper panel) does not show a significant difference between the treatments. Body-weights on the day of brain extraction (Day 67; lower panel) shows that CIS treated mice eventually reached comparable weights to VEH, CAF, and CIS+CAF treated mice. (**D**) Diagram showing Arm-A (start arm), Arm-B, and Arm-C in the Y-maze test for assessment of working memory. (**E**) Y-maze working memory analysis shows that VEH, CAF, and CIS+CAF treated mice exhibited higher % spontaneous alternation levels in comparison to CIS treated mice. (**F**) Assessment of distance traveled in the Y-maze shows that both VEH and CAF treated mice had significantly more exploratory activity when compared to CIS treated mice. There was no difference in distance traveled between CIS+CAF and CIS treated mice. (**B**): RM 2-way ANOVA, Dunnett’s *post-hoc*. n = 7-10 mice/treatment group. (**C**): 1-way ANOVA, Dunnett’s *post-hoc*. n = 7-10 mice/treatment group. (**E**): 1-way ANOVA, Holm-Sidak’s *post-hoc*. n = 7-10 mice/treatment group. (**F**): 1-way ANOVA, Tukey’s *post-hoc*. n = 7-10 mice/treatment group. Not significant (ns): *P* > 0.05, *: *P* < 0.05, **: *P* < 0.01, ***: *P* < 0.001. Results are reported in mean ± SEM.


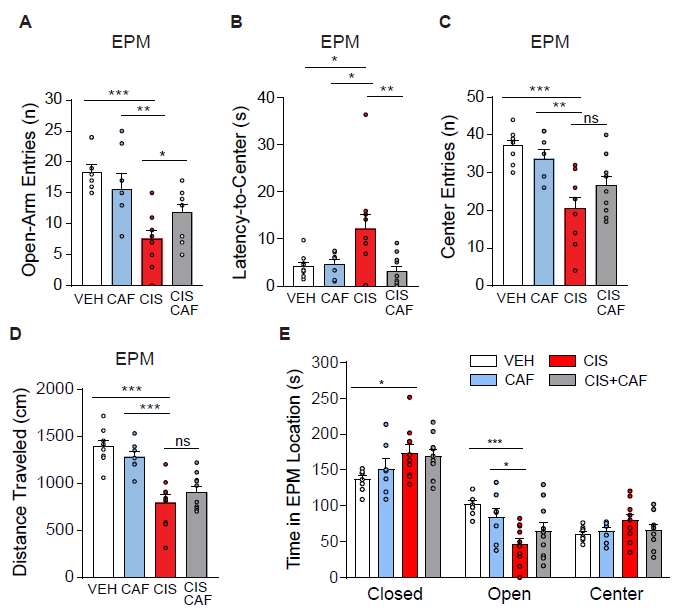


**Supplementary Fig. 5. Effect of caffeine (37.5 mg/kg) on elevated plus maze performance.** (**A**) Elevated plus maze (EPM) analysis of open-arm entries by vehicle-alone (VEH) treated mice given oral gavage of water, mice given oral gavage of caffeine-alone (CAF; 37.5 mg/kg, *p.o.*), and mice given oral gavage of caffeine plus injections of cisplatin (CIS+CAF). VEH, CAF and CIS+CAF cohorts had significantly more entries to this exposed section of the maze, when compared to CIS treated mice. (**B**) Analysis of latency to enter the center location showed that VEH, CAF, and CIS+CAF treated mice waited less time to enter this exposed section of the maze when compared to CIS treated mice. (**C**) Analysis of EPM center entry frequency shows that VEH and CAF treated mice had significantly more entries to this exposed section of the maze, when compared to CIS treated mice, although we did not detect a significant difference in center entries between CIS+CAF and CIS treated mice. (**D**) Assessment of distance traveled in the EPM shows that both VEH and CAF treated mice had significantly more exploratory activity when compared to CIS treated mice, although there was no difference in distance traveled between CIS+CAF and CIS treated mice. (**E**) Time spent in EPM location shows that CIS treated mice spent more time in the closed arms when compared to VEH controls. Similarly, CIS treated mice spent significantly less time in the open-arms when compared to VEH and CAF controls. There was no difference in time spent in maze location between CIS+CAF and CIS-alone treated mice. (**A, C**): 1-way ANOVA, Holm-Sidak’s *post-hoc*. n = 7-10 mice/treatment group. (**B, D**): 1-way ANOVA, Dunnett’s *post-hoc*. n = 7-10 mice/treatment group. (**E**): 2-way ANOVA, Tukey’s *post-hoc*. n = 7-10 mice/treatment group. Not significant (ns): *P* > 0.05, *: *P* < 0.05, **: *P* < 0.01, ***: *P* < 0.001. Results are reported in mean ± SEM.

**References**

Albani, S.H., Mchail, D.G., and Dumas, T.C. (2014). Developmental studies of the hippocampus and hippocampal-dependent behaviors: insights from interdisciplinary studies and tips for new investigators. *Neurosci Biobehav Rev.* 43**,** 183-190. doi: 10.1016/j.neubiorev.2014.04.009.

Ardais, A.P., Borges, M.F., Rocha, A.S., Sallaberry, C., Cunha, R.A., and Porciuncula, L.O. (2014). Caffeine triggers behavioral and neurochemical alterations in adolescent rats. *Neuroscience.* 270**,** 27-39. doi: 10.1016/j.neuroscience.2014.04.003.

Arshadi, C., Gunther, U., Eddison, M., Harrington, K.I.S., and Ferreira, T.A. (2021). SNT: a unifying toolbox for quantification of neuronal anatomy. *Nat Methods.* 18**,** 374-377. doi: 10.1038/s41592-021-01105-7.

Bonati, M., Jiritano, L., Bortolotti, A., Gaspari, F., Filippeschi, S., Puidgemont, A., and Garattini, S. (1985). Caffeine distribution in acute toxic response among inbred mice. *Toxicol Lett.* 29**,** 25-31. doi: 10.1016/0378-4274(85)90195-x.

Breglio, A.M., Rusheen, A.E., Shide, E.D., Fernandez, K.A., Spielbauer, K.K., Mclachlin, K.M., Hall, M.D., Amable, L., and Cunningham, L.L. (2017). Cisplatin is retained in the cochlea indefinitely following chemotherapy. *Nat Commun.* 8**,** 1654. doi: 10.1038/s41467-017-01837-1.

Callizot, N., Andriambeloson, E., Glass, J., Revel, M., Ferro, P., Cirillo, R., Vitte, P.A., and Dreano, M. (2008). Interleukin-6 protects against paclitaxel, cisplatin and vincristine-induced neuropathies without impairing chemotherapeutic activity. *Cancer Chemother Pharmacol.* 62**,** 995-1007. doi: 10.1007/s00280-008-0689-7.

Chiu, G.S., Boukelmoune, N., Chiang, A.C.A., Peng, B., Rao, V., Kingsley, C., Liu, H.L., Kavelaars, A., Kesler, S.R., and Heijnen, C.J. (2018). Nasal administration of mesenchymal stem cells restores cisplatin-induced cognitive impairment and brain damage in mice. *Oncotarget.* 9**,** 35581-35597. doi: 10.18632/oncotarget.26272.

Chiu, G.S., Maj, M.A., Rizvi, S., Dantzer, R., Vichaya, E.G., Laumet, G., Kavelaars, A., and Heijnen, C.J. (2017). Pifithrin-mu Prevents Cisplatin-Induced Chemobrain by Preserving Neuronal Mitochondrial Function. *Cancer Res.* 77**,** 742-752. doi: 10.1158/0008-5472.CAN-16-1817.

Cognato, G.P., Agostinho, P.M., Hockemeyer, J., Muller, C.E., Souza, D.O., and Cunha, R.A. (2010). Caffeine and an adenosine A(2A) receptor antagonist prevent memory impairment and synaptotoxicity in adult rats triggered by a convulsive episode in early life. *J Neurochem.* 112**,** 453-462. doi: 10.1111/j.1471-4159.2009.06465.x.

Daley-Yates, P.T., and Mcbrien, D.C. (1984). Enhancement of cisplatin nephrotoxicity by probenecid. *Cancer Treat Rep.* 68**,** 445-446. doi.

Demaria, M., O'leary, M.N., Chang, J., Shao, L., Liu, S., Alimirah, F., Koenig, K., Le, C., Mitin, N., Deal, A.M., Alston, S., Academia, E.C., Kilmarx, S., Valdovinos, A., Wang, B., De Bruin, A., Kennedy, B.K., Melov, S., Zhou, D., Sharpless, N.E., Muss, H., and Campisi, J. (2017). Cellular Senescence Promotes Adverse Effects of Chemotherapy and Cancer Relapse. *Cancer Discov.* 7**,** 165-176. doi: 10.1158/2159-8290.CD-16-0241.

Du, F. (2019). Golgi-Cox Staining of Neuronal Dendrites and Dendritic Spines With FD Rapid GolgiStain Kit. *Curr Protoc Neurosci.* 88**,** e69. doi: 10.1002/cpns.69.

Duarte, J.M., Carvalho, R.A., Cunha, R.A., and Gruetter, R. (2009). Caffeine consumption attenuates neurochemical modifications in the hippocampus of streptozotocin-induced diabetic rats. *J Neurochem.* 111**,** 368-379. doi: 10.1111/j.1471-4159.2009.06349.x.

Ferreira, T.A., Blackman, A.V., Oyrer, J., Jayabal, S., Chung, A.J., Watt, A.J., Sjostrom, P.J., and Van Meyel, D.J. (2014). Neuronal morphometry directly from bitmap images. *Nat Methods.* 11**,** 982-984. doi: 10.1038/nmeth.3125.

George, K.C., Hebbar, S.A., Kale, S.P., and Kesavan, P.C. (1999). Caffeine protects mice against whole-body lethal dose of gamma-irradiation. *J Radiol Prot.* 19**,** 171-176. doi: 10.1088/0952-4746/19/2/306.

Gill, J.S., and Windebank, A.J. (1998). Cisplatin-induced apoptosis in rat dorsal root ganglion neurons is associated with attempted entry into the cell cycle. *J Clin Invest.* 101**,** 2842-2850. doi: 10.1172/JCI1130.

Gondal, J.A., Preuss, H.G., Swartz, R., and Rahman, A. (1993). Comparative pharmacological, toxicological and antitumoral evaluation of free and liposome-encapsulated cisplatin in rodents. *Eur J Cancer.* 29A**,** 1536-1542. doi: 10.1016/0959-8049(93)90290-v.

Hinton, D.J., Andres-Beck, L.G., Nett, K.E., Oliveros, A., Choi, S., Veldic, M., and Choi, D.S. (2019). Chronic caffeine exposure in adolescence promotes diurnal, biphasic mood-cycling and enhanced motivation for reward in adult mice. *Behav Brain Res.* 370**,** 111943. doi: 10.1016/j.bbr.2019.111943.

Hughes, R.N. (2004). The value of spontaneous alternation behavior (SAB) as a test of retention in pharmacological investigations of memory. *Neurosci Biobehav Rev.* 28**,** 497-505. doi: 10.1016/j.neubiorev.2004.06.006.

Hussaini, S.M., Jun, H., Cho, C.H., Kim, H.J., Kim, W.R., and Jang, M.H. (2013). Heat-induced antigen retrieval: an effective method to detect and identify progenitor cell types during adult hippocampal neurogenesis. *J Vis Exp.* doi: 10.3791/50769.

Hussien, M., and Yousef, M.I. (2022). Impact of ginseng on neurotoxicity induced by cisplatin in rats. *Environ Sci Pollut Res Int.* 29**,** 62042-62054. doi: 10.1007/s11356-021-16403-y.

Kaster, M.P., Machado, N.J., Silva, H.B., Nunes, A., Ardais, A.P., Santana, M., Baqi, Y., Muller, C.E., Rodrigues, A.L., Porciuncula, L.O., Chen, J.F., Tome, A.R., Agostinho, P., Canas, P.M., and Cunha, R.A. (2015). Caffeine acts through neuronal adenosine A2A receptors to prevent mood and memory dysfunction triggered by chronic stress. *Proc Natl Acad Sci U S A.* 112**,** 7833-7838. doi: 10.1073/pnas.1423088112.

Le, X., and Hanna, E.Y. (2018). Optimal regimen of cisplatin in squamous cell carcinoma of head and neck yet to be determined. *Ann Transl Med.* 6**,** 229. doi: 10.21037/atm.2018.05.10.

Ma, J., Huo, X., Jarpe, M.B., Kavelaars, A., and Heijnen, C.J. (2018). Pharmacological inhibition of HDAC6 reverses cognitive impairment and tau pathology as a result of cisplatin treatment. *Acta Neuropathol Commun.* 6**,** 103. doi: 10.1186/s40478-018-0604-3.

Moreira-Pais, A., Ferreira, R., and Gil Da Costa, R. (2018). Platinum-induced muscle wasting in cancer chemotherapy: Mechanisms and potential targets for therapeutic intervention. *Life Sci.* 208**,** 1-9. doi: 10.1016/j.lfs.2018.07.010.

Morrison, H.W., and Filosa, J.A. (2013). A quantitative spatiotemporal analysis of microglia morphology during ischemic stroke and reperfusion. *J Neuroinflammation.* 10**,** 4. doi: 10.1186/1742-2094-10-4.

Nakagawa, H., Fujita, T., Kubo, S., Tokiyoshi, K., Yamada, M., Kanayama, T., Hagiwara, Y., Nakanomyo, H., and Hiraoka, M. (1996). Difference in CDDP penetration into CSF between selective intraarterial chemotherapy in patients with malignant glioma and intravenous or intracarotid administration in patients with metastatic brain tumor. *Cancer Chemother Pharmacol.* 37**,** 317-326. doi: 10.1007/s002800050391.

Ness, K.K., Krull, K.R., Jones, K.E., Mulrooney, D.A., Armstrong, G.T., Green, D.M., Chemaitilly, W., Smith, W.A., Wilson, C.L., Sklar, C.A., Shelton, K., Srivastava, D.K., Ali, S., Robison, L.L., and Hudson, M.M. (2013). Physiologic frailty as a sign of accelerated aging among adult survivors of childhood cancer: a report from the St Jude Lifetime cohort study. *J Clin Oncol.* 31**,** 4496-4503. doi: 10.1200/JCO.2013.52.2268.

Oliveros, A., Wininger, K., Sens, J., Larsson, M.K., Liu, X.C., Choi, S., Faka, A., Schwieler, L., Engberg, G., Erhardt, S., and Choi, D.S. (2017). LPS-induced cortical kynurenic acid and neurogranin-NFAT signaling is associated with deficits in stimulus processing during Pavlovian conditioning. *J Neuroimmunol.* 313**,** 1-9. doi: 10.1016/j.jneuroim.2017.09.010.

Oliveros, A., Yoo, K.H., Rashid, M.A., Corujo-Ramirez, A., Hur, B., Sung, J., Liu, Y., Hawse, J.R., Choi, D.S., Boison, D., and Jang, M.H. (2022). Adenosine A2A receptor blockade prevents cisplatin-induced impairments in neurogenesis and cognitive function. *Proc Natl Acad Sci U S A.* 119**,** e2206415119. doi: 10.1073/pnas.2206415119.

Onaolapo, A.Y., and Onaolapo, O.J. (2015). Caffeine's influence on object recognition and working-memory in prepubertal mice and its modulation by gender. *Pathophysiology.* 22**,** 223-230. doi: 10.1016/j.pathophys.2015.09.001.

Pandolfo, P., Machado, N.J., Kofalvi, A., Takahashi, R.N., and Cunha, R.A. (2013). Caffeine regulates frontocorticostriatal dopamine transporter density and improves attention and cognitive deficits in an animal model of attention deficit hyperactivity disorder. *Eur Neuropsychopharmacol.* 23**,** 317-328. doi: 10.1016/j.euroneuro.2012.04.011.

Perse, M. (2021). Cisplatin Mouse Models: Treatment, Toxicity and Translatability. *Biomedicines.* 9. doi: 10.3390/biomedicines9101406.

Seal, A.D., Bardis, C.N., Gavrieli, A., Grigorakis, P., Adams, J.D., Arnaoutis, G., Yannakoulia, M., and Kavouras, S.A. (2017). Coffee with High but Not Low Caffeine Content Augments Fluid and Electrolyte Excretion at Rest. *Front Nutr.* 4**,** 40. doi: 10.3389/fnut.2017.00040.

Seale, T.W., Johnson, P., Carney, J.M., and Rennert, O.M. (1984). Interstrain variation in acute toxic response to caffeine among inbred mice. *Pharmacol Biochem Behav.* 20**,** 567-573. doi: 10.1016/0091-3057(84)90306-x.

Szturz, P., Wouters, K., Kiyota, N., Tahara, M., Prabhash, K., Noronha, V., Adelstein, D., Van Gestel, D., and Vermorken, J.B. (2019). Low-Dose vs. High-Dose Cisplatin: Lessons Learned From 59 Chemoradiotherapy Trials in Head and Neck Cancer. *Front Oncol.* 9**,** 86. doi: 10.3389/fonc.2019.00086.

Ta, L.E., Low, P.A., and Windebank, A.J. (2009). Mice with cisplatin and oxaliplatin-induced painful neuropathy develop distinct early responses to thermal stimuli. *Mol Pain.* 5**,** 9. doi: 10.1186/1744-8069-5-9.

Vorhees, C.V., and Williams, M.T. (2006). Morris water maze: procedures for assessing spatial and related forms of learning and memory. *Nat Protoc.* 1**,** 848-858. doi: 10.1038/nprot.2006.116.

Walf, A.A., and Frye, C.A. (2007). The use of the elevated plus maze as an assay of anxiety-related behavior in rodents. *Nat Protoc.* 2**,** 322-328. doi: 10.1038/nprot.2007.44.

Wang, S., Prizment, A., Thyagarajan, B., and Blaes, A. (2021). Cancer Treatment-Induced Accelerated Aging in Cancer Survivors: Biology and Assessment. *Cancers (Basel).* 13. doi: 10.3390/cancers13030427.

Yoo, K.H., Tang, J.J., Rashid, M.A., Cho, C.H., Corujo-Ramirez, A., Choi, J., Bae, M.G., Brogren, D., Hawse, J.R., Hou, X., Weroha, S.J., Oliveros, A., Kirkeby, L.A., Baur, J.A., and Jang, M.H. (2021). Nicotinamide Mononucleotide Prevents Cisplatin-Induced Cognitive Impairments. *Cancer Res.* 81**,** 3727-3737. doi: 10.1158/0008-5472.CAN-20-3290.

Young, K., and Morrison, H. (2018). Quantifying Microglia Morphology from Photomicrographs of Immunohistochemistry Prepared Tissue Using ImageJ. *J Vis Exp.* doi: 10.3791/57648.
